# Joint-RPCA: domain-aware multi-omics integration for systems microbiology

**DOI:** 10.1038/s44320-026-00236-3

**Published:** 2026-08-18

**Authors:** Bianca Cordazzo Vargas, Cameron Martino, Amanda Hazel Dilmore, Jessica L Metcalf, Zachary M Burcham, Leo Lahti, Aituar Bektanov, Tuomas Borman, Veikko Salomaa, Teemu Niiranen, Aki S Havulinna, Rachel Gregor, Stav Eyal, Michael M Meijler, Itzhak Mizrahi, Se Jin Song, Andrew Bartko, Pieter C Dorrestein, James T Morton, Daniel McDonald, Rob Knight, Liat Shenhav

**Affiliations:** 1 Institute for Systems Genetics, New York University Grossman School of Medicine, New York, NY USA; 2 Leaven Foods, San Diego, CA USA; 3 Department of Pediatrics, University of California San Diego, La Jolla, CA USA; 4 Biomedical Sciences Graduate Program, University of California San Diego, La Jolla, CA USA; 5 Department of Animal Sciences, Colorado State University, Fort Collins, CO USA; 6 Department of Microbiology, University of Tennessee, Knoxville, TN USA; 7 Department of Computing, FI-20014 University of Turku, Turku, Finland; 8 Department of Public Health, Finnish Institute for Health and Welfare, Helsinki, Finland; 9 Department of Internal Medicine, Turku University Hospital and University of Turku, Turku, Finland; 10 Institute for Molecular Medicine Finland, FIMM- HiLIFE, Helsinki, Finland; 11 Department of Chemical Engineering and Applied Chemistry, University of Toronto, Toronto, ON Canada; 12 National Institute of Biotechnology in the Negev, Ben-Gurion University of the Negev, Be’er Sheva, Israel; 13 Department of Life Sciences, Ben-Gurion University of the Negev, Be’er Sheva, Israel; 14 Department of Chemistry, Ben-Gurion University of the Negev, Be’er Sheva, Israel; 15 Center for Microbiome Innovation, University of California San Diego, La Jolla, CA USA; 16 Collaborative Mass Spectrometry Innovation Center, Skaggs School of Pharmacy and Pharmaceutical Sciences, University of California San Diego, La Jolla, CA USA; 17 Department of Pharmacology, School of Medicine, University of California San Diego, La Jolla, CA USA; 18 Department of Computer Science and Engineering, University of California, San Diego, La Jolla, CA USA; 19 Department of Bioengineering, University of California San Diego, San Diego, La Jolla, CA USA; 20 Department of Microbiology, New York University Grossman School of Medicine, New York, NY USA; 21 Department of Obstetrics and Gynecology, New York University Grossman School of Medicine, New York, NY USA; 22 Department of Computer Science, Courant Institute of Mathematical Sciences, New York University, New York, NY USA

**Keywords:** Computational Biology, Microbiology, Virology & Host Pathogen Interaction

## Abstract

Integrating multi-omics data is essential for microbiome research, as microbial communities are shaped by and respond to interdependent processes, including taxonomic composition, metabolite production and utilization, and gene expression. However, accurately capturing ecosystem-wide patterns across these modalities is statistically challenging due to differences in scale, sparsity, and compositionality. While a growing number of multi-omics methods have emerged, they differ in their mathematical objectives and modeling assumptions, which in turn shape how biological patterns are represented and interpreted. This underscores the need for tools that explicitly account for the statistical properties of microbial ecosystems. Here, we present Joint Robust Principal Component Analysis (Joint-RPCA), a method designed with these statistical properties in mind and broadly applicable to multi-omics settings with similar challenges. Built on the OptSpace matrix completion framework, Joint-RPCA assumes an underlying shared low-rank structured component across modalities to identify shared variation and cross-modal associations from matched samples. Within this setting and under these statistical assumptions, Joint-RPCA showed stronger performance than the benchmarked general-purpose methods in phenotype separation and feature association tasks, achieving up to sixfold improvement in classification accuracy and over 100-fold faster runtimes. Applied to real-world datasets, including the Integrative Human Microbiome Project (iHMP), mammalian gut microbiomes, and decomposition studies, Joint-RPCA reveals replicable and interpretable multi-omic patterns, offering a scalable and domain-aware solution for systems-level microbiome analysis. Joint-RPCA is available in both Python (https://github.com/biocore/gemelli) and R (https://bioconductor.org/packages/mia).

## Synopsis

Joint-RPCA is a novel context-aware method that integrates multi-omics microbiome data through low-rank matrix completion, enabling discovery of robust cross-modal associations and improved performance over general multi-omics approaches in accuracy, interpretability, and runtime.

- Joint-RPCA jointly factorizes multiple omic layers, revealing shared low-rank structures across microbial, metabolomic, and transcriptomic data.
- Cross-modal feature associations were recovered, including validated microbe-metabolite interactions, demonstrating robustness to sparsity and compositionality in microbiome data.
- Replicable multi-omic disease markers and ecosystem-wide patterns missed by single-omic analyses were identified across independent IBD cohorts, human decomposition data, and mammalian gut microbiomes.
- Joint-RPCA demonstrates up to sixfold improvement in classification accuracy and over 100-fold faster runtime relative to existing multi-omics integration methods.

## Abstract (web summary)

Joint-RPCA is a novel context-aware method that integrates multi-omics microbiome data through low-rank matrix completion, enabling discovery of robust cross-modal associations and improved performance over general multi-omics approaches in accuracy, interpretability, and runtime.

## Graphical abstract

**Figure:**
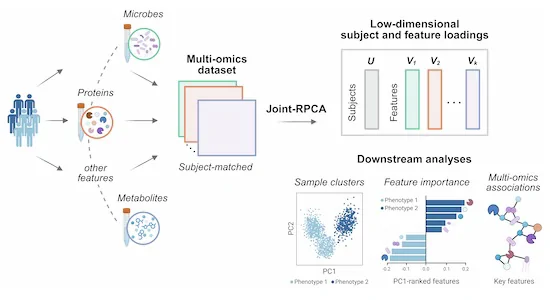

**Figure:**
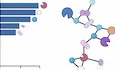

## Introduction

Microbial ecosystems play a crucial role in shaping ecological and physiological outcomes, either by directly influencing biological processes or by serving as biomarkers of underlying system states, whether host-related or environmental. These influences manifest through multiple dimensions, including microbial abundances, metabolite production and consumption, and microbial gene expression. Capturing these complex interactions requires integrating diverse data types, as different aspects of the microbial ecosystem are measured using distinct technologies.

A multi-omics approach, where multiple layers of biological data, such as microbiome sequencing, metabolomics, and gene expression, are combined, offers a more comprehensive representation of microbial function and host interactions (Lloyd-Price et al, 2019; Jansson and Baker, 2016). However, integrating these datasets presents significant challenges due to fundamental differences in data structure and properties. These challenges include compositionality (Pawlowsky-Glahn et al, 2015), where microbial sequencing data reflects relative rather than absolute abundances; sparsity (Hamady and Knight, 2009; Morton et al, 2017), where many measurements contain missing or zero values; and scale differences (Potvin and Roff, 1993), where different omics technologies generate data at varying magnitudes and units. As a result, researchers often analyze each data modality in isolation, missing opportunities to leverage interdependencies across datasets. At the same time, these challenges are not unique to microbiome data, but also arise in other high-dimensional biological datasets, including transcriptomic and proteomic data, suggesting broader applicability for methods designed to address them.

One common approach to exploring data sampled from microbial communities is by performing dimensionality reduction (such as principal coordinates analysis or PCoA) on a distance matrix composed of pairwise sample dissimilarities. This allows unsupervised global differences within a dataset to be observed. When working with multiple data modalities from the same microbial ecosystem, this approach can be applied separately to each dataset. However, doing so treats the data layers as independent and fails to account for the inherent correlations between these modalities that were sampled from the same ecosystem. One example is the production of specific metabolites by specific bacteria (e.g., GABA produced by human *Bacteroides* species (Strandwitz et al, 2019) or regulated by *Akkermansia muciniphila* (Olson et al, 2018). An alternative to analyzing multi-omics layers independently is the use of supervised methods, which focus on building models based on assumed relationships between data modalities. However, these assumptions may be overly simplified as the true relationships are frequently unknown (Ruiz-Perez et al, 2021). Further, the output of these methods may not directly allow clustering of phenotypes by microbial community properties (Mallick et al, 2019).

To address these challenges, we developed joint robust principal component analysis (joint-RPCA), an unsupervised framework for multi-omics dimensionality reduction in microbiome data that produces both a traditional beta-diversity analysis and a differential feature abundance assessment. Joint-RPCA assumes that the input data matrices can be approximated by a low-rank structure; that is, represented by a small number of latent factors, yielding a structured component amid higher-rank variation (e.g., biological and technical noise).

In this context, “low-rank” means the dominant structured component is well-approximated by a small number of latent factors. While this can correspond to capturing a large share of structured variance, the signal may still be small relative to total variation when embedded in high-rank variation. In microbiome settings, the relevant phenotype-associated effect may be low-dimensional (often effectively rank-1 in a two-group contrast) yet explain only a small fraction of total variance, because it is embedded in substantial high-rank biological and technical heterogeneity. Accordingly, we use ‘low-dimensional signal’ to refer to the dimensionality of the latent phenotype factor, not the fraction of total variance it explains. Biologically, this suggests that a limited set of microbial factors, such as taxa abundances or metabolite levels, captures a low-dimensional phenotype-associated signal. Here, variance refers to differences in feature abundance (e.g., microbial taxa, gene expression, or metabolites) across individuals and across data modalities (Martino et al, 2019). The dimensionality reduction performed by Joint-RPCA leverages cross-modality relationships to recover a low-rank approximation of the joint structured signal shared across modalities, reducing high-rank noise and increasing statistical power.

**Figure 1:**
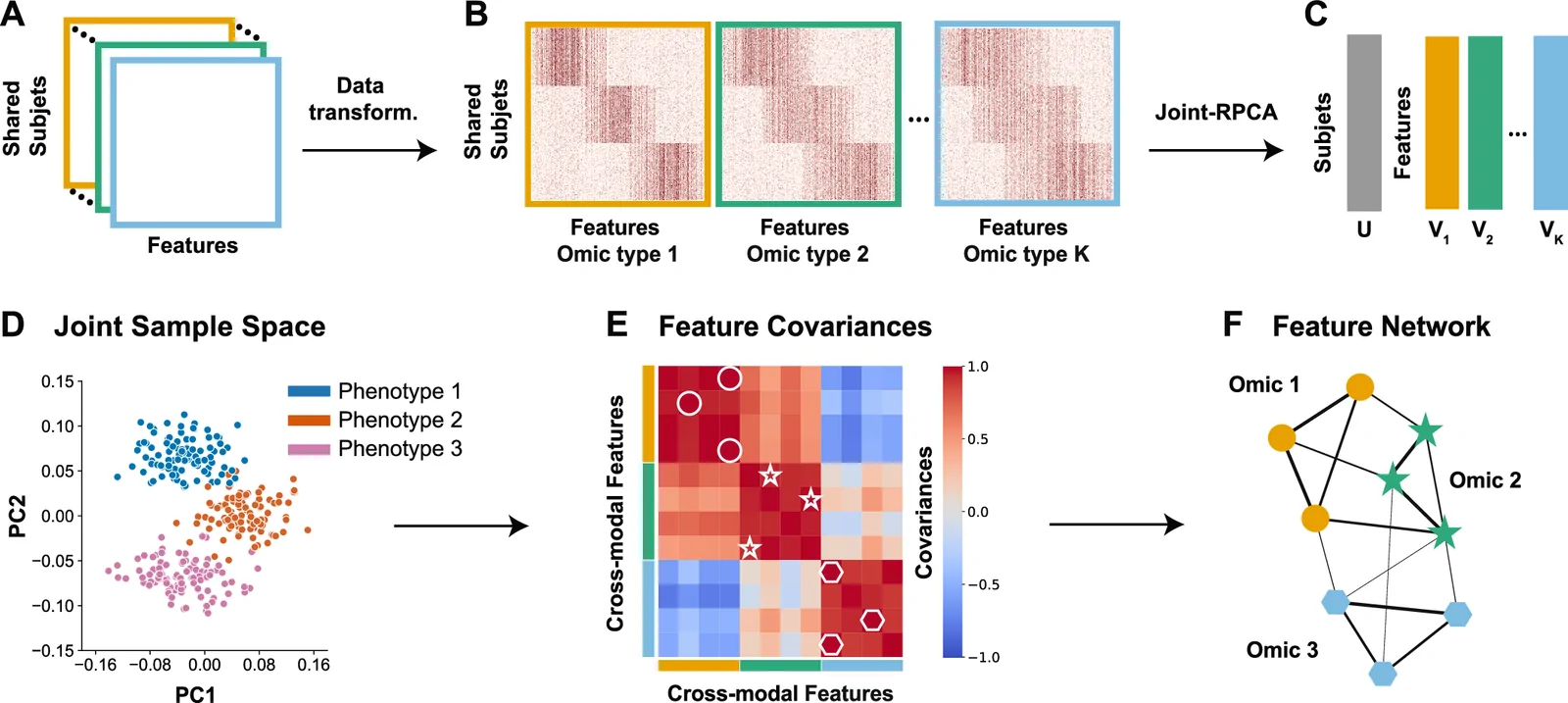
Overview of the Joint-RPCA algorithm. Here, we use synthetic simulated data to illustrate the Joint-RPCA framework, highlighting both its inputs and outputs. In this toy multi-omics dataset, we simulated three sample groups (representing different phenotypes) per omic type. (**A**) Joint-RPCA takes as input K data modalities (feature matrices; here K = 3) measured across the same subjects. (**B**) These matrices are preprocessed to ensure comparability (e.g., by adjusting scale and variance). (**C**) The transformed matrices are jointly decomposed into a shared sample space (U), and distinct, modality-specific feature spaces $\left({V}_{i}\right)$. (**D**) Shown here are the first two principal components of the shared sample space for the synthetic data; in real datasets, such separation is not guaranteed and depends on the strength of the underlying biological signals. (**E**) A feature-feature covariance matrix is computed across modalities to estimate interactions within the ecosystem. (**F**) A feature network can be constructed to visualize the strength of feature interactions associated with a phenotype of interest, where nodes represent features and edges depict intra- and inter-modal relationships. For simplicity, only a small subset of features is illustrated for each modality. Circles, triangles, and hexagons represent features from Omics 1–3, respectively.

In the first step of Joint-RPCA, each data modality is transformed to account for sparsity, missing data points, compositionality and the potentially different scales of the omics’ measurements (Gloor et al, 2017; Quinn et al, 2019) (Fig. 1A,B, Methods). Next, a joint dimensionality reduction of the different data modalities is performed using OptSpace, a matrix completion algorithm that computes a low-rank approximation of each data matrix via singular value decomposition optimized on a local manifold (Keshavan et al, 2009). The sample space is jointly estimated across all modalities, while the feature space is estimated individually, within each modality. Joint factorization of these different data modalities yields distinct vectors for subjects (defined here as “*U*”; known as the scores matrix, shared across all data modalities) and features (defined here as “*V*_*i*_*”; i* ∈ {1,...,*K*}, known as the loadings matrix, unique per modality Fig. 1C). Because these vectors are unit-scaled, their entries can be ranked to reflect their association with the underlying patterns in the data. From here on, we will refer to the ordering of these vectors as “rankings” (e.g., *V* = “feature rankings”).

**Figure 2:**
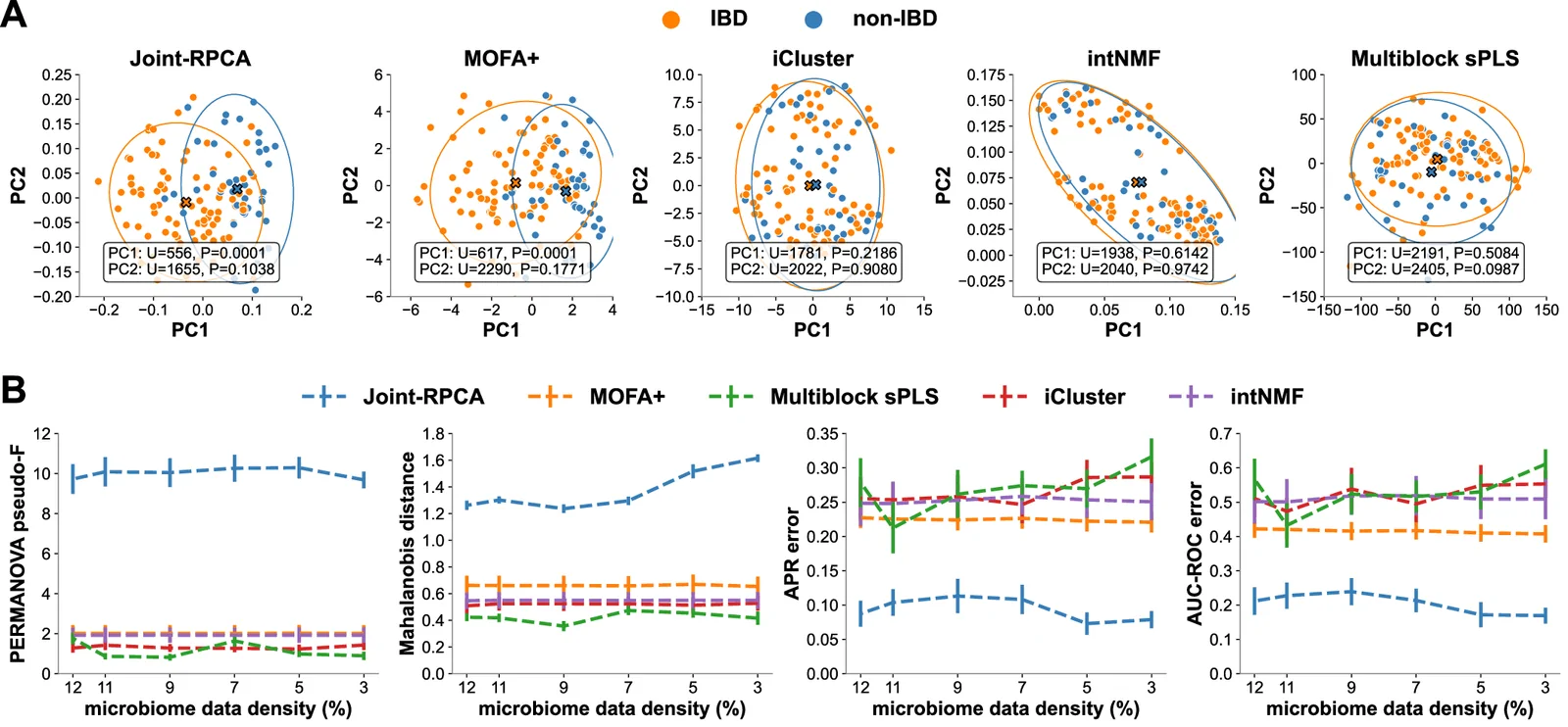
Joint-RPCA shows stronger phenotype separation than benchmarked general-purpose dimensionality-reduction methods across multiple data modalities in the setting studied. (**A**) Joint-RPCA and multi-omics methods (MOFA+, iCluster, intNMF, and multiblock sPLS from mixOmics) applied to metabolomics, proteomics, viromics, and microbiome metagenomics and metatranscriptomics from the iHMP data. Points are colored by IBD (*n* = 91, orange) and non-IBD (*n* = 44, blue) diagnosis, and the cluster centroids are highlighted. Wilcoxon rank-sum test statistics and associated *p* values are shown. Methods limited to single modality plotting or analysis (RPCA, Aitchison, and Bray-Curtis) are shown separately in Appendix Fig. S1. (**B**) Performance evaluation on data-driven simulations with increasing data sparsity (12% observed density to 3% induced, x-axis), assessed using PERMANOVA pseudo-F statistic, Mahalanobis distance between phenotype centroids, as well as Random Forest classification error (area under the precision-recall curve (APR) error and area under the receiver operating characteristic curve (AUC-ROC) error) (y-axis) as complementary measures of phenotype differentiation. Comparison includes Joint-RPCA (blue), MOFA+ (orange), unsupervised sPLS from mixOmics (green), iCluster (red), and intNMF (purple). Error bars show the standard error of the mean across *n* = 10 cross-validation folds, with each fold corresponding to a distinct train-test split. Source data are available online for this figure.

Joint-RPCA combines two analytical approaches into a single framework: multi-omics dimensionality reduction and the estimation of cross-modal feature interactions. It takes as input *K* data matrices (*K* is unbounded), each representing distinct feature types collected concurrently across a shared set of samples. The output includes: (1) a joint, low-dimensional representation of the samples across these *K* data matrices (i.e., sample loadings, Fig. 1D); (2) feature loadings for each omic type, indicating the contribution of individual features to different axes of variation; and (3) a denoised version of the feature-feature covariance matrix, capturing interactions across data modalities (Fig. 1E,F).

**Figure 3:**
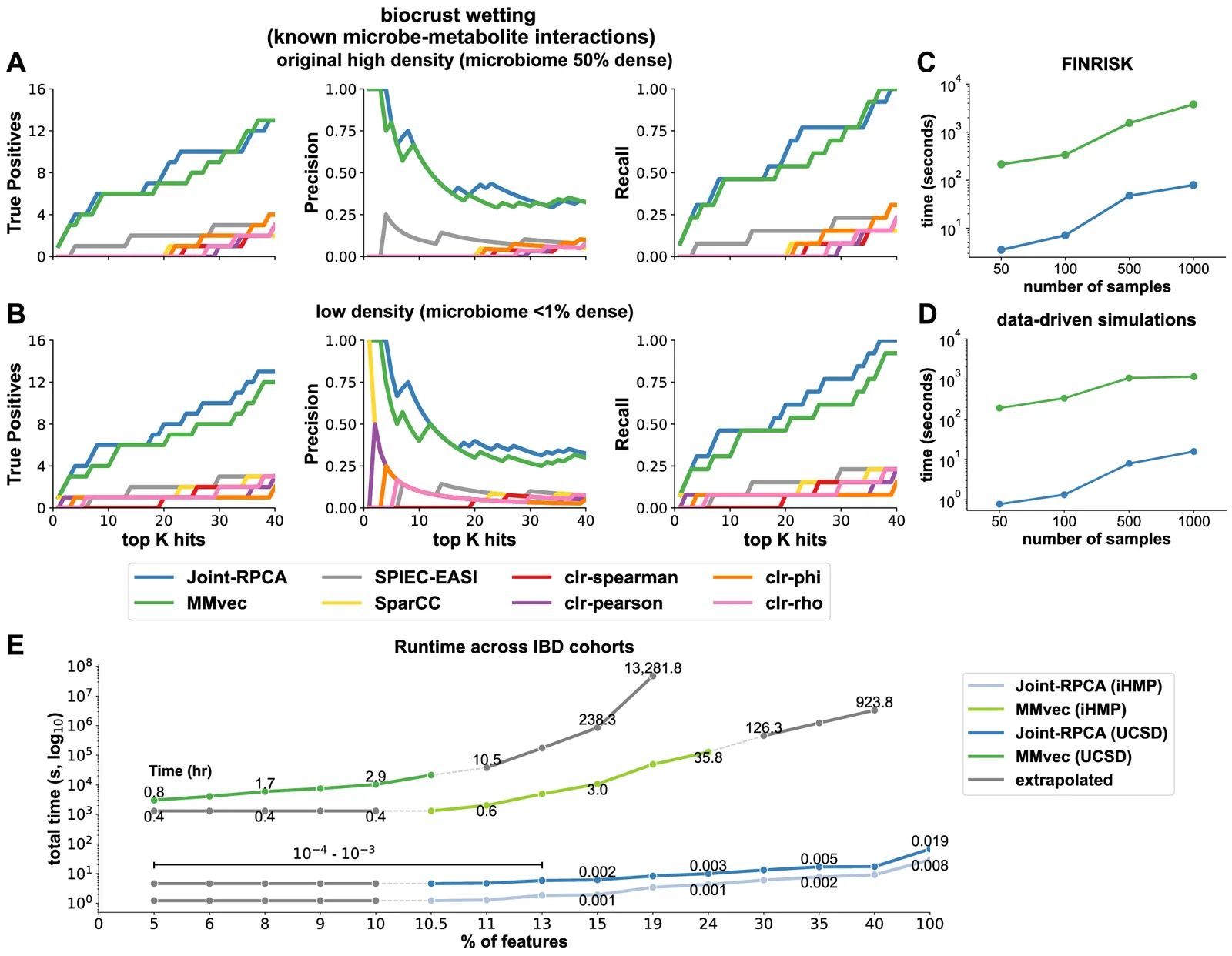
Joint-RPCA substantially reduces runtime (over 100x) while maintaining or exceeding accuracy. (**A**, **B**) Benchmarking of multi-omics feature-feature interactions of experimentally validated molecules of *M. vaginatus* following biocrust wetting events evaluated through true positives (left-column), precision (middle-column), and recall (left-columns) (y-axes) among the top K ranked hits (x-axes) on (**A**) dense and (**B**) in silico subsampled sparse data. Methods compared are Joint-RPCA (blue), MMvec (green), SPIEC-EASI (gray), SparCC (yellow), and Spearman (red), Pearson (purple), phi (orange), and rho (pink) correlation metrics on CLR-transformed data with imputation using table averages. (**C**, **D**) Comparison of runtime in seconds (x-axis) between MMvec (green) and Joint-RPCA (blue) across different numbers of samples (y-axis) for (**C**) real and (**D**) simulated data. (**E**) Runtime comparisons using two independent IBD cohorts to illustrate the significant time cost incurred by MMvec during replicability studies. Source data are available online for this figure.

To evaluate its performance, we benchmarked Joint-RPCA against widely used microbiome dimensionality reduction methods, such as PCoA with Bray-Curtis (Bray and Curtis, 1957) and Aitchison distance (Aitchison, 1983), as well as RPCA (Martino et al, 2019), each applied separately to single data modalities (Appendix Fig. S1). Additionally, we compared Joint-RPCA to state-of-the-art multi-omics methods not designed specifically for microbiome data, including MOFA+ (Argelaguet et al, 2020) (the latest version of MOFA), iClusterPlus (Shen et al, 2009) (the latest version of iCluster), intNMF (Chalise and Fridley, 2017), and multiblock sPLS from mixOmics (Rohart et al, 2017) (Fig. 2A,B). Joint-RPCA showed stronger performance than the other benchmarked methods across increasing levels of data sparsity, with classification accuracy improving by up to sixfold in this benchmark setting (Fig. 2B). These results suggest that, under these conditions, Joint-RPCA more consistently recovers shared cross-modal structure linked to phenotype separation than either single-modality microbiome methods or general multi-omics approaches. Using experimentally validated interactions between microbes and metabolites, we further benchmarked Joint-RPCA against correlation-based approaches and MMvec (Morton et al, 2019), a method specifically designed to estimate microbe-metabolite interactions. We demonstrate that Joint-RPCA successfully recovers experimentally validated relationships between two critical components of microbial ecosystems, microbial abundances and metabolite levels, while reducing runtime by a factor of 100 compared to MMvec (Fig. 3A–E).

To summarize, Joint-RPCA facilitates two important analytical goals simultaneously: estimating shared patterns across datasets and identifying how features from each dataset relate to each other. This allows researchers to examine microbial, metabolic, and gene expression data jointly, rather than in isolation, uncovering biological relationships that would otherwise remain hidden or require multiple independent analyses. Unlike existing tools, Joint-RPCA is designed to handle the sparsity and compositionality characteristics of microbiome data and to scale efficiently to more than two omic types, offering a unified and interpretable view of a biological ecosystem.

## Results

### Evaluation of Joint-RPCA using in silico simulations and experimental data

First, we evaluate Joint-RPCA’s ability to differentiate sample groups, recover low-dimensional (phenotype) signal, identify cross-modal feature interactions, and achieve computational efficiency using both in silico simulations and experimental data that reflect common characteristics of microbiome datasets. We benchmark Joint-RPCA against widely used multi-omics tools for dimensionality reduction and inference of microbe-metabolite interactions.

#### Joint-RPCA differentiates sample groups in the joint sample space

To demonstrate the utility of Joint-RPCA in differentiating sample groups within host-associated microbial ecosystems, based on a low-dimensional (phenotype) signal, we first applied it to data-driven simulated datasets comprising five modalities and two underlying phenotypic groups. Simulations were generated using real metabolomics, proteomics, viromics, metagenomics, and metatranscriptomic data from the Integrative Human Microbiome Project (iHMP) (Lloyd-Price et al, 2019; Integrative HMP (iHMP) Research Network Consortium, 2014), while varying microbiome data sparsity in the metagenomics modality through sequencing depth, as previously described (Äijö et al, 2018; Martino et al, 2021) (Methods). This dataset was chosen due to the strong phenotypic separation between individuals with and without inflammatory bowel disease (IBD) and the distinct signatures of IBD across individual modalities (Lloyd-Price et al, 2019). We also note that the signal distinguishing these phenotypes has previously been shown to arise from a low-dimensional signal (Lloyd-Price et al, 2019). These simulations allowed direct assessment of each method’s robustness to information loss in a single modality, mimicking the high sparsity often encountered in microbiome data, while maintaining the overall biological structure of the data.

We compared Joint-RPCA’s performance to commonly used dimensionality reduction methods designed for microbiome data, which perform single-modality analysis, including PCoA with Bray-Curtis (Bray and Curtis, 1957) and Aitchison distance (Aitchison, 1983), as well as RPCA (Martino et al, 2019), each applied independently to individual modalities (Appendix Fig. S1). Additionally, we benchmarked Joint-RPCA against general-purpose multi-omics methods not specifically designed for microbiome data, including MOFA+ (Argelaguet et al, 2020), iClusterPlus (Shen et al, 2009), intNMF (Chalise and Fridley, 2017), and multiblock sPLS (Rohart et al, 2017) (Fig. 2A,B). For the data-driven simulations, we split the data into train-test splits for Joint-RPCA and MOFA+, which are capable of projecting unseen samples into the learned latent space (Fig. 2B; Appendix Fig. S2A). Joint-RPCA utilizes these projections as an internal metric of model fit and generalizability, as well as for user-driven parameter tuning (Methods). To evaluate phenotype separation in the inferred latent spaces, we used complementary metrics: Wilcoxon rank-sum tests comparing the distribution of sample groups along individual latent dimensions, PERMANOVA pseudo-F statistics computed from pairwise distances in the latent space, Mahalanobis distance between-group centroids, and Random Forest classification of disease status using the inferred subject loadings.

Of note, although MOFA+ learns a single joint latent factor model, projections of unseen data are by design modality-specific. We therefore evaluated Random Forest classifiers on each modality-specific projection separately and reported the unweighted mean across projections as a summary of out-of-sample generalization (Appendix Fig. S2B). The remaining benchmarked methods were evaluated using their standard implementations, as described in Methods. Across the benchmark setting studied, Joint-RPCA showed stronger recovery of IBD-associated structure (Fig. 2B).

Random Forest classification of disease status (IBD vs. non-IBD) using the sample loadings estimated by each method showed that Joint-RPCA consistently yielded lower classification error across simulated microbiome data densities (i.e., the proportion of nonzero values in the metagenomics modality; Fig. 2B). When all methods were trained on the full dataset without train-test splits (Fig. 2A), Joint-RPCA and MOFA+ both produced low-dimensional representations showing substantial IBD vs. non-IBD separation. These differences became more apparent in the data-driven simulation setting with induced sparsity and explicit train-test evaluation (Fig. 2B), where Joint-RPCA yielded more stable out-of-sample performance.

Joint-RPCA also showed greater Mahalanobis distances between IBD and non-IBD centroids and higher PERMANOVA pseudo-F statistics across simulated microbiome densities (Fig. 2B; Appendix Table S1). Although some curves in Fig. 2B appear nearly flat across simulated microbiome densities, the underlying metrics do vary with density. For some methods, however, these changes are modest after averaging across cross-validation folds (and for MOFA+, across modality-specific projections) and are visually compressed by the y-axis scale. Overall, these simulations indicate that Joint-RPCA more consistently recovers phenotype-associated structure in this benchmark setting.

Next, we assessed how well each method recovered a low-dimensional phenotype-associated signal. Although many features differ between IBD and non-IBD samples, prior work has shown that much of this variation is organized into a coherent low-dimensional signal (Lloyd-Price et al, 2019). To evaluate whether the methods recovered this shared structure, we compared the top-ranked features identified by Joint-RPCA and MOFA+ along the principal component or factor most strongly associated with IBD diagnosis, focusing on the metabolomics modality because it yielded the best predictive performance for MOFA+ (Appendix Fig. S2B). Across train-test splits and simulated metagenomic data densities, Joint-RPCA more consistently identified a similar set of metabolomic features, showing a 60–70% median overlap, whereas MOFA+ exhibited less than 30% overlap (Appendix Fig. S2C). This consistency indicates that Joint-RPCA more stably recovers the shared low-dimensional phenotype-associated signal across train/test splits, whereas MOFA+ tends to select features that vary more across training sets.

To complement these findings, we designed in silico synthetic simulations to test Joint-RPCA’s ability to recover a shared low-dimensional phenotype-associated signal across data modalities under controlled conditions (Methods). These simulations were designed to construct sparse, compositional multi-omics datasets with traceable signals, enabling a direct assessment of a method’s ability in detecting known induced signals. Across three simulated microbiome-like datasets with varying numbers of features with induced signals (*n* = 5–60), Joint-RPCA consistently differentiated samples with induced signals from those without, regardless of signal strength, whereas iClusterPlus, intNMF, and multiblock sPLS showed variable performance, and MOFA+ improved at higher levels of introduced signal (Appendix Fig. S3A; Appendix Table S2).

Moreover, Joint-RPCA reliably identified the specific features carrying the induced signal, ranking them among its top loadings across folds, whereas MOFA+ exhibited less consistent rankings, particularly when signals were weak (Appendix Fig. S3B,C). Together, our data-driven and in silico synthetic simulations show that Joint-RPCA can robustly recover a shared low-dimensional phenotype-associated signal across modalities in sparse and compositional settings.

Next, we performed simulations to assess how the effect size between sample groups and data dimensionality influences Joint-RPCA’s performance. Here, effect size is defined as the degree of feature overlap between groups within a modality (Methods). When there is zero overlap, groups are perfectly separable (large effect size), while greater overlap reduces the distinct signals distinguishing these groups (small effect size). Data dimensionality is defined as the ratio of features to samples. A schematic of simulations with varying effect sizes is provided in Appendix Fig. S4A. We generated datasets with 1, 10, and 100 times as many features as samples, while increasing feature overlap, effectively decreasing effect sizes. Joint-RPCA was applied independently to each simulated dataset and to pairwise combinations of datasets with different effect sizes at a fixed dimensionality. PERMANOVA pseudo-F statistics on sample loadings showed that Joint-RPCA’s discriminative performance remained stable when the effect size in at least one modality is moderate to high (Appendix Fig. S4B), but that it was sensitive to pairs of datasets with overall low effect sizes (not shown). We also evaluated stability and reproducibility of results by running Joint-RPCA five times on pairwise combinations of effect sizes at each dimensionality, confirming that the resulting sample and feature loadings were consistent across runs (Appendix Fig. S4C). In these simulations, the number of samples is not the primary determinant of Joint-RPCA’s performance; rather, performance was more strongly influenced by the magnitude of between-group differences (i.e., effect size).

Overall, in both data-driven and in silico simulations, Joint-RPCA recovered shared low-dimensional phenotype-associated structure more consistently than the benchmarked general-purpose multi-omics methods under sparse, compositional data conditions. These performance differences should be interpreted in this specific context rather than as a general ranking of integration tools. The benchmarked methods differ in their modeling assumptions and analytic objectives, which likely contributes to their variable performance under these conditions (see Appendix Supplementary Note for a detailed comparison of multi-omics methods).

#### Joint-RPCA captures cross-modal feature covariance

Beyond dimensionality reduction and uncovering the patterns of shared variation across data modalities, another important output of Joint-RPCA is the feature-feature covariance matrix, which represents the estimated relationships between features across different data modalities. For example, interactions between microbes and the metabolites they release and consume. This covariance matrix is derived from the feature loadings obtained through the joint factorization, making it a denoised representation of the covariance structure across all data modalities (Methods). This matrix has dimensions of *n*×*n* (all input features by all input features) and can be interpreted as an adjacency matrix of a multipartite graph, enabling the generation of a multi-omics feature network. In this context, a value in the matrix represents an edge between two features (i.e., nodes), and the weight of the edge is the covariance or association between the features.

To evaluate the utility of the Joint-RPCA feature-feature covariance matrix in capturing biologically meaningful relationships across data modalities, we applied it to a study of biological soil crusts (biocrusts) wetting events (Swenson et al, 2018). Biocrusts are thin layers of living organisms and organic material that form on soil surfaces in arid, semi-arid, and other environments with limited vegetation cover. In this study, metabolites taken up or secreted by several biocrust isolates, including *Microcoleus vaginatus*(Baran et al, 2015), were compared with metabolite profiles from related environmental taxa and in situ samples (Swenson et al, 2018).

This dataset was chosen for two main reasons. First, ~70% of the microbe-metabolite relationships examined following the wetting event were experimentally validated (Swenson et al, 2018), although some relationships contradicted those observed in culture studies (Baran et al, 2015) (specifically for *M. vaginatus)*. This combination of validation and discrepancies provided an ideal opportunity to test whether Joint-RPCA could not only recapitulate known interactions but also resolve previously unexplained inconsistencies observed when using simple Spearman correlations. This made the dataset an ideal test case for evaluating the ability of Joint-RPCA to uncover biologically relevant relationships across multi-omics data in a well-characterized system.

Second, this dataset was previously used to demonstrate the utility of a computational method called MMvec (Morton et al, 2019), which was specifically designed to quantify interactions between microbes and metabolites. MMvec successfully recapitulated experimentally validated interactions and resolved the observed discrepancies. Given the concerns raised about the accuracy of MMvec compared to linear correlations applied after a centered log-ratio (CLR) transformation, as proposed by Quinn and Erb (Quinn and Erb, 2021), we compare Joint-RPCA to both approaches. Specifically, we benchmark Joint-RPCA against MMvec and linear correlations with CLR transformation (using imputation of the tables’ means through propr (Quinn et al, 2017)), as well as other designated methods such as SPIEC-EASI (Kurtz et al, 2015) and SparCC (Friedman and Alm, 2012), adhering to the methodology outlined by Morton et al (Morton et al, 2021). Furthermore, to examine the stability of Joint-RPCA at high sparsity levels, traditionally a major influence on accuracy (Quinn and Erb, 2021; Morton et al, 2021), the microbiome data were subsampled from 50% dense to 10% and 1% dense.

Using Joint-RPCA, we found that all metabolites released by the *M. vaginatus* isolate in culture studies showed positive covariance values with *M. vaginatus* from the in situ biocrust samples (relative abundances across samples are provided in Appendix Table S3). Additionally, the covariance between *M. vaginatus* and each of these metabolites was higher than the average covariance between *M. vaginatus* and all other metabolites following biocrust wetting. These metabolites were among the top 40 co-varying metabolites associated with *M. vaginatus* out of 85 molecules in total. Notably, this finding remained robust across all levels of sparsity (Appendix Table S4). More broadly, we observed that, regardless of sparsity level, Joint-RPCA and MMvec exhibited significantly higher true-positive rates, precision, and recall compared to other metrics used to infer feature relationships (Fig. 3A,B; Appendix Fig. S5). Importantly, the feature-feature covariance produced by Joint-RPCA remained consistent across different numbers of features and samples, highlighting its ability to deliver robust results even in datasets with large feature spaces and small sample sizes (*n* « *p*; Appendix Fig. S4C).

#### Joint-RPCA enables fast and scalable analysis

A key advantage of Joint-RPCA over the current state-of-the-art method, MMvec, is its speed, which directly enhances reproducibility and biological discovery. To assess this, we performed runtime experiments using data-driven simulations and FINRISK (Borodulin et al, 2018), one of the largest multi-omics microbiome studies to date, comprising 7167 individuals aged 25 to 74 years, with shotgun metagenomic samples of the microbiome produced from stool, in tandem with metabolomics produced from blood samples from the same subjects (Salosensaari et al, 2021). Joint-RPCA reduced runtime by approximately a factor of 100 compared to MMvec (Fig. 3C,D; Appendix Table S5). This improvement stems from fundamental differences in model design. MMvec estimates the conditional probability p(x|r) between metabolite abundances (x) and microbial reads (r) on a per-read basis. While this avoids the typical *n* < *p* problem, since the number of reads often exceeds the number of samples, it comes at a computational cost, as runtime scales linearly with the number of reads. In contrast, Joint-RPCA resolves the *n* < *p* challenge by construction, enabling it to operate on a per-sample basis. As a result, its runtime scales with the number of samples rather than the number of reads, yielding substantial speed gains in large datasets. Consequently, Joint-RPCA can simultaneously estimate thousands of samples and features per omic type on the order of minutes, where MMvec may take anything upward of days.

Time efficiency is critical not only for processing multiple omic types simultaneously but also for reproducibility across independent cohorts, an essential requirement for robust biological discoveries. Unlike MMvec, which is limited to analyzing only two omic types at a time, Joint-RPCA integrates multiple omic layers simultaneously, significantly improving the scalability of discovery and validation. This capability is particularly important for large, multi-cohort studies, where MMvec’s pairwise approach substantially increases computational time, making systematic replication challenging in practice.

To illustrate this, we conducted additional runtime experiments using two independent IBD cohorts: the iHMP cohort (five omic types, 68 patients) (Lloyd-Price et al, 2019) and the UCSD cohort (three omic types, 146 patients) (Mills et al, 2022). We progressively increased the number of features in Joint-RPCA and MMvec until reaching the full dataset. Because MMvec requires pairwise analysis of all omic combinations, certain pairs were excluded due to prohibitively long runtimes, which we instead extrapolated (Methods). In these cohort-scale benchmarks, Joint-RPCA successfully processed thousands of samples and features across omic types and cohorts within seconds to minutes, whereas MMvec required days to analyze only subsets of the data (Fig. 3E; Dataset EV1). In summary, by enabling rapid, scalable, and simultaneous multi-omics integration, Joint-RPCA not only enhances reproducibility but can also accelerate the identification of biologically meaningful interactions, making it a powerful tool for large-scale, multi-cohort studies.

### Application of Joint-RPCA to real data

We next applied Joint-RPCA to three real datasets to demonstrate the utility of joint dimensionality reduction for multi-omics data across different contexts.

#### Joint-RPCA reveals reproducible IBD signatures across independent datasets

We first analyzed multi-omics data from the Integrative Human Microbiome Project (iHMP), which includes 135 matched metabolomics, proteomics, viromics, metagenomics, and metatranscriptomics samples from 68 patients diagnosed with or without inflammatory bowel disease (IBD) (Lloyd-Price et al, 2019). Using Joint-RPCA, we successfully distinguished IBD (Ulcerative Colitis (UC) & Crohn’s Disease (CD)) from non-IBD subjects across all omic layers (PERMANOVA pseudo-*F* = 17.04, *P* = 0.0005) along PC1 (Fig. 2A).

**Figure 4:**
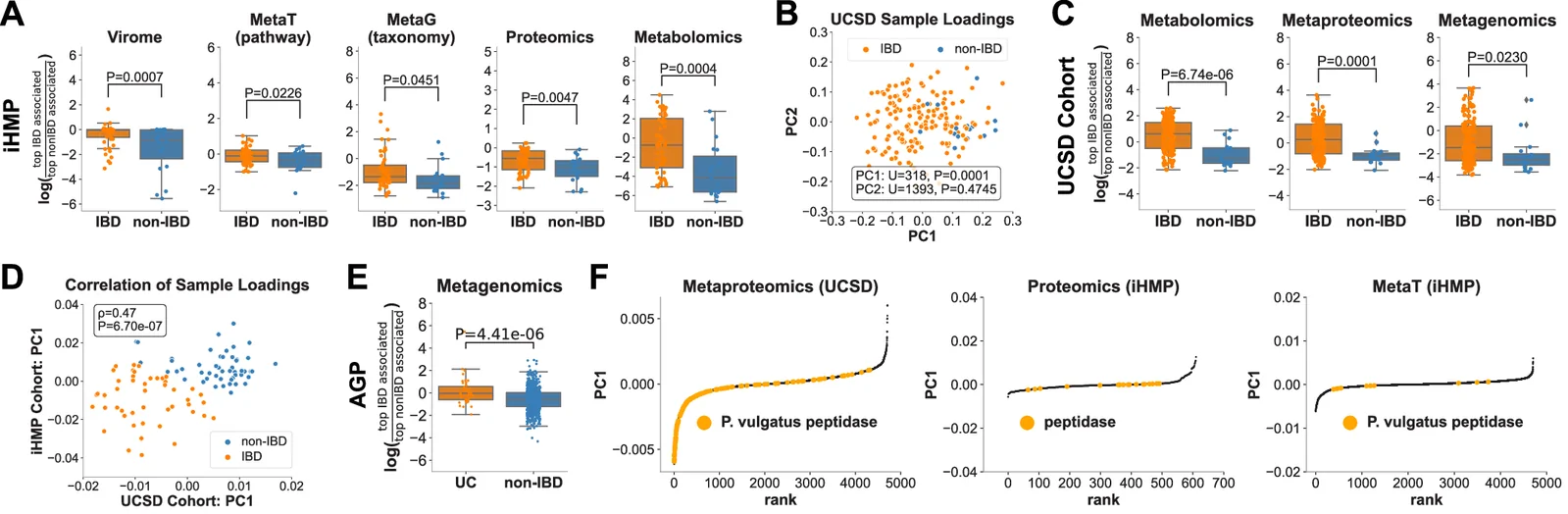
Joint-RPCA identifies reproducible IBD-associated patterns across independent cohorts. (**A**) Joint-RPCA applied to five data modalities (metabolomics, proteomics, viromics, metagenomics and metatranscriptomics) from the iHMP study separates IBD (*n* = 49, orange) from non-IBD (*n* = 19, blue) subjects along the first axis (y-axis). An independent t-test shows that the log-ratio of the top and bottom Joint-RPCA PC1-ranked features within each omic significantly differentiates IBD from non-IBD samples (x-axis). For each box plot, the center line indicates the median, the box bounds indicate the interquartile range (25th–75th percentiles), and the whiskers extend to the most extreme data points within 1.5× the interquartile range. (**B**) Joint-RPCA applied to three data modalities (metabolomics, proteomics, and metagenomics) from the UCSD cohort separates subjects by IBD (orange) vs. non-IBD (blue) diagnosis along PC1 (y-axis). Wilcoxon rank-sum test; statistics and associated *p* values are shown. (**C**) The log-ratio of the top and bottom Joint-RPCA PC1-ranked features within each omic significantly separates IBD (*n* = 157, orange) from non-IBD (*n* = 16, blue) subjects in the UCSD cohort, as assessed by independent *t*-test. For each box plot, the center line indicates the median, the box bounds indicate the interquartile range (25th–75th percentiles), and the whiskers extend to the most extreme data points within 1.5× the interquartile range. (**D**) Significant correlation (*r* = 0.47, *P* = 6.70 × 10^−7^) between IBD-associated metagenomics feature rankings in the iHMP and UCSD datasets. (**E**) The log-ratio of the top and bottom Joint-RPCA PC1 metagenomics features shared by the iHMP and UCSD cohorts significantly separates IBD (*n* = 39) and non-IBD subjects (*n* = 819; assessed by independent *t*-test) in a third independent validation dataset, which includes samples from the American Gut Project (AGP). For each box plot, the center line indicates the median, the box bounds indicate the interquartile range (25th–75th percentiles), and the whiskers extend to the most extreme data points within 1.5× the interquartile range. (**F**) The ranking (x-axis) of peptidases (orange) associated with *Phocaeicola vulgatus* in metaproteomic UCSD data (left), proteomics data from the iHMP cohort (middle), and metatranscriptomic iHMP data (right) along Joint-RPCA axis 1 (y-axis), showing enrichment in IBD-diagnosed subjects. Source data are available online for this figure.

Next, to validate the differentiation of samples by IBD diagnosis along PC1, we selected the top and bottom features from each omic type, ranked by this PC, and examined their log-ratios. Joint-RPCA revealed a consistent and significant separation between IBD and non-IBD subjects across all omic types in the same direction (Independent *t*-test *P* < 0.05 for all comparisons), whereas RPCA, when applied independently to each omic type, did not capture this consistent shared structure (Fig. 4A; Appendix Fig. S6). Specifically, RPCA was unable to distinguish IBD from non-IBD using metatranscriptomic and metabolomics data alone. However, Joint-RPCA successfully identified metatranscriptomic and metabolomic features that differentiate these two phenotypic groups by leveraging the “context” of proteomics, viromics, and metagenomics, demonstrating its ability to integrate multiple omic layers to reveal disease-associated patterns. Notably, Joint-RPCA identified several cross-modal features previously recognized as key markers in the iHMP study, including urobilin (metabolomics) and *Klebsiella spp*. (metagenomics) (Lloyd-Price et al, 2019). Specifically, three urobilin metabolites ranked among the top six metabolites associated with non-IBD diagnosis, while *Klebsiella pneumoniae* ranked among the top 15% of taxa associated with IBD, aligning with iHMP findings (Lloyd-Price et al, 2019).

We further validated our findings using an independent dataset that includes 173 matched metabolomics, metaproteomics, and metagenomics samples from a different cohort (i.e., the “UCSD cohort”) of 146 subjects diagnosed with or without IBD (specifically UC) (Mills et al, 2022). Consistent with our findings in the iHMP dataset, Joint-RPCA significantly separated IBD from non-IBD subjects across all omics (PERMANOVA pseudo-*F* = 12.99, *P* = 0.0005) along PC1 (Fig. 4B). Next, we selected the top and bottom features from each omic type, ranked by PC1, which distinguishes IBD from non-IBD, and examined their log-ratios. These log-ratios produced significant separations between IBD and non-IBD subjects across all omic types (independent *t*-test *P* < 0.05 for all comparisons), in the same direction as observed in the iHMP dataset, reinforcing the robustness and reproducibility of Joint-RPCA across independent cohorts (Fig. 4C).

To directly compare findings across both studies, we correlated the rankings of IBD-associated metagenomic features between the iHMP and UCSD cohorts and found a significant correlation of 0.47 (Spearman Rho *P* = 6.70 ×  10^−^⁷; Fig. 4D). Notably, the top-ranked features identified by Joint-RPCA in both cohorts were distinct from those identified independently by RPCA (Appendix Fig. S6). We further validated our findings using another dataset consisting exclusively of metagenomic data, which included 40 IBD subjects from a third cohort (Mills et al, 2022) and 824 non-IBD, non-IBS controls from the American Gut Project (McDonald et al, 2018) (Methods). When comparing the log-ratios of metagenomic features identified in the iHMP and UCSD cohorts, we observed a significant separation between IBD and non-IBD subjects in this third independent dataset (independent t-test *P* < 0.001), consistent in both direction and effect with the previous two cohorts (Fig. 4E).

Notably, across all three datasets, *Phocaeicola vulgatus* (previously *Bacteroides vulgatus*) emerged as the top bacterial species associated with IBD. This species has been experimentally linked to UC/CD through its production of proteases, which Joint-RPCA also identified as highly associated in both metatranscriptomics and proteomics (Mills et al, 2022) (Fig. 4F). In summary, Joint-RPCA uncovered a robust microbial signature of IBD, replicated across three independent datasets, while also identifying multi-omic relationships that were overlooked by single-omic analyses (Fig. 4A; Appendix Fig. S6).

#### Joint-RPCA identifies universal microbial networks in human decomposition

To further evaluate the performance of Joint-RPCA, we applied it to a multi-omic dataset from Burcham et al, which characterized microbial dynamics during human cadaver decomposition using 16S and 18S rRNA sequencing, shotgun metagenomics, and metabolomics (Burcham et al, 2024). This dataset includes samples from skin and soil swabs associated with 36 willed-body donors collected daily during the first 21 days of decomposition across three distinct forensic anthropological facilities, representing different geographic locations, climates, and seasons.

**Figure 5:**
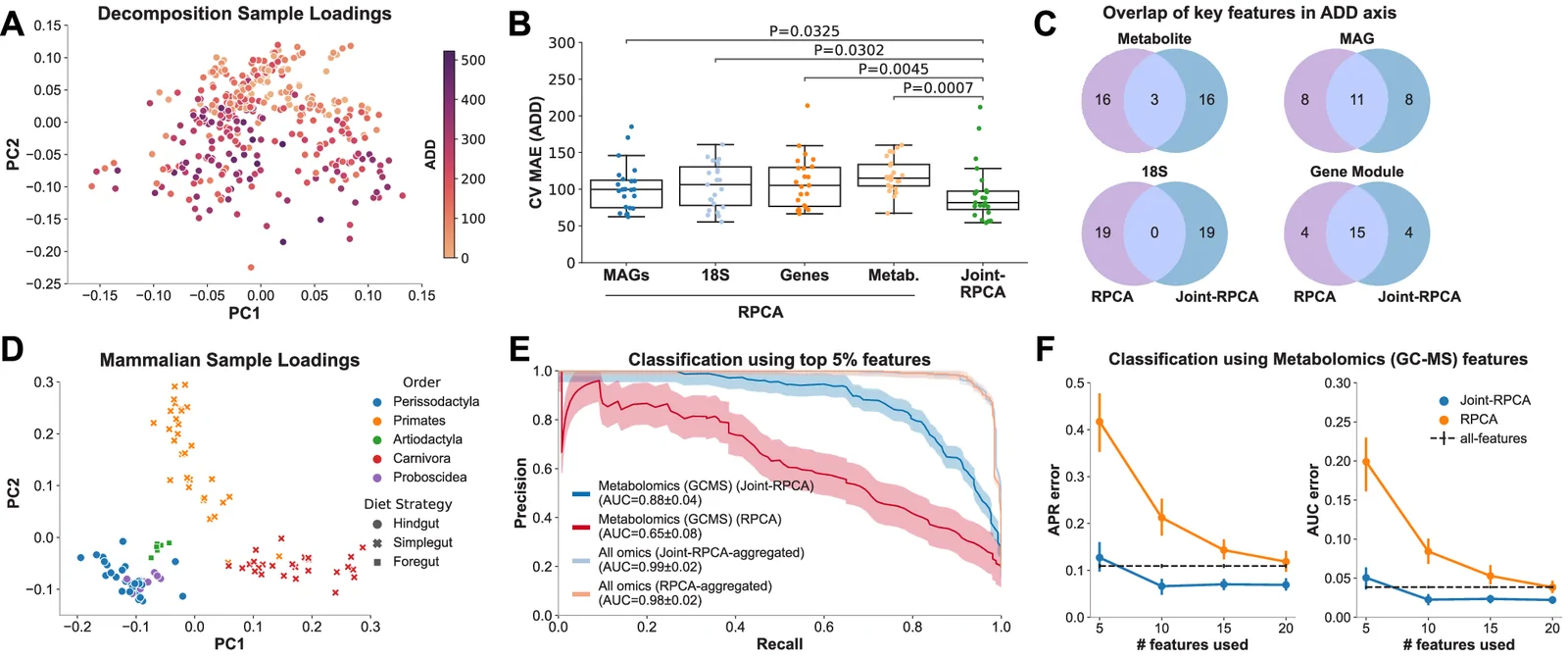
Integrating ecosystem-wide data in microbial communities using Joint-RPCA improves host-phenotype resolution and enables the identification of key features across modalities. (**A**–**C**) Case study on human cadaver decomposition. (**A**) Joint-RPCA applied to four data modalities (16S and 18S rRNA sequencing, shotgun metagenomics, and metabolomics) captures the progression of accumulated degree days (ADD) along PC2. (**B**) Random Forest prediction of ADD samples, comparing ordination results from individual omic analysis with RPCA to Joint-RPCA, shows that Joint-RPCA yields significantly lower cross-validated mean absolute error (CV MAE) than individual omics (Wilcoxon signed-rank test; *p* values are shown). For each box plot, the center line indicates the median, the box bounds indicate the interquartile range (25th–75th percentiles), and the whiskers extend to the most extreme data points within 1.5× the interquartile range. For each method, each point represents a cross-validation fold corresponding to a distinct train-test split (*n* = 23). (**C**) Comparison of the top features associated with advanced decomposition stages resulting from individual omic analysis (RPCA) and Joint-RPCA reveals that RPCA misses a large proportion of metabolites and all bacterial features in the universal decomposer network revealed by Burcham et al (Burcham et al, 2024). (**D**–**F**) Case study on mammalian gut microbiomes. (**D**) Joint-RPCA applied to five data modalities (16S rRNA sequencing, GC-MS, semi-polar metabolomics, and both positive- and negative-mode lipidomics) identified host taxonomy and digestive strategy as key drivers of variation, demonstrating its ability to extract biologically meaningful patterns across multiple omic layers. (**E**) Metabolomics (GC-MS) data were used to classify host taxonomy and digestive strategy using a Random Forest classifier, with performance measured by the area under the precision-recall curve. Using the top 5% of features ranked by Joint-RPCA (blue) resulted in higher classification accuracy than features ranked by RPCA alone (red), indicating that Joint-RPCA prioritizes biologically relevant metabolic signatures. (**F**) When using a small number of features (5 to 20, x-axis), Joint-RPCA-ranked metabolomics features (blue) consistently achieved higher predictive accuracy for host taxonomy and digestive strategy for mammalian samples (*n* = 101), as measured by average precision-recall (y-axis), than RPCA applied solely to metabolomics data (orange). This demonstrates that Joint-RPCA effectively selects informative features, improving classification performance even with a limited feature set. Error bars show the standard deviation across *n* = 10 cross-validation folds, with each fold corresponding to a distinct train-test split. Source data are available online for this figure.

Joint-RPCA identified an axis of variation (PC2) that distinguished samples by stage of decomposition as well as accumulated degree days (ADD), a temperature-based timeline of decomposition (Burcham et al, 2024) (Fig. 5A), and uncovered associated features across multiple omic types along this axis. These linked features were used to construct a universal microbial decomposer network, enriched for taxa and metabolites associated with advanced stages of decomposition (Appendix Fig. S7). Notably, due to strong environmental differences between facilities, identifying shared patterns across locations was particularly challenging. However, Joint-RPCA was the only method that successfully leveraged all omic types simultaneously, enabling the identification of a universal decomposer network shared across facilities.

To assess how Joint-RPCA successfully identified this universal decomposer network, we compared top features associated with advanced decomposition stages identified by Joint-RPCA versus individual RPCA feature loadings. RPCA applied independently to each omic type failed to detect key microbial eukaryotic features from 18S rRNA and captured only a subset of relevant features from other omic types (Fig. 5C). In contrast, Joint-RPCA uniquely identified important features, including *Yarrowia* and *Candida* from 18S and *Thiopseudomonas alkaliphila* (prev. *Oblitimonas alkaliphila*) (Burcham, 2024) from metagenomics, which were highly correlated in the universal decomposer network (Burcham et al, 2024). As proposed by Burcham et al, these findings may reflect inter-domain interactions (e.g., fungi–bacteria), where *Yarrowia* and *Candida* break down lipids and proteins, which are then utilized by *T. alkaliphila* (Burcham et al, 2024; Ashe et al, 2021). The highly interconnected role of *T. alkaliphila* in the decomposer network further underscores Joint-RPCA’s ability to capture both inter- and intra-domain interactions, particularly inter-domain relationships, which were not captured by RPCA applied to individual omic types.

To further highlight the value of joint factorization of multi-omics data, we compared the results obtained from Joint-RPCA to those from RPCA performed separately for each omic. First, we confirmed that the PC2 axis from Joint-RPCA clearly captures the progression of ADD (Fig. 5A), a trend that is less consistent in the individual RPCA ordinations (Appendix Fig. S8). Additionally, we found that the axis that best represents changes in ADD varied across omic types, as shown by the Kruskal–Wallis H-statistic (Dataset EV2). Second, we compared the prediction accuracy when using the sample loadings from either Joint-RPCA or individual RPCA as Random Forest features. Our results showed that Joint-RPCA yielded lower cross-validated mean absolute error than individual RPCA in predicting ADD, with CV mean absolute errors significantly lower than all individual omics (Wilcoxon signed-rank test *P* < 0.05 compared to MAGs and 18S, *P* < 0.01 compared to metagenomics, and *P* < 0.001 compared to metabolomics) (Fig. 5B).

Overall, these findings demonstrate how Joint-RPCA’s ability to integrate multiple omic types simultaneously enables the identification of biologically relevant, cross-omic interactions that are not readily detected by single-omic approaches. By capturing shared patterns across diverse environments, Joint-RPCA provides a powerful framework for multi-omic integration, particularly in complex ecological studies.

#### Joint-RPCA enhances integration and classification in mammalian gut microbiomes

To further evaluate the utility of Joint-RPCA, we applied it to a multi-omic dataset that characterized the metabolic content of mammalian gut microbiomes as a direct window into ecosystem function (Gregor et al, 2022). This dataset includes fecal samples from 25 mammalian species and incorporates five omic types: 16S rRNA sequencing, gas chromatography–mass spectrometry (GC-MS), semi-polar metabolomics, and both positive and negative lipidomics. By applying Joint-RPCA to all five omic types simultaneously, we found that host taxonomy and digestive strategy were the primary factors driving sample separation, consistent with findings from the original study (Fig. 5D). This result highlights Joint-RPCA’s ability to identify biologically relevant patterns across multiple omic layers while preserving key ecological signals.

We next investigated how Joint-RPCA resolves signals from omic types with weaker discriminatory power. Among the five modalities, GC-MS metabolomics showed the weakest performance in classifying samples by host taxonomy and digestive strategy when analyzed independently (Appendix Fig. S9). However, Joint-RPCA effectively amplified the signal from GC-MS, revealing meaningful biological variation that was otherwise difficult to detect (Fig. 5E). Notably, although the GC-MS alone exhibited limited discriminatory power, a subset of its features emerged as some of the most informative along all axes of variation in the joint analysis, showing that Joint-RPCA can enhance detection of signals from weaker modalities.

To quantify the impact of Joint-RPCA on classification performance, we trained a Random Forest classifier using the top 5% of features from all omic types (*n* = 2250) and compared the results when features were ranked by either Joint-RPCA or independent RPCA. Both methods performed comparably (AUC-ROC = 0.99 ± 0.02 for Joint-RPCA, AUC-ROC = 0.98 ± 0.02 for independent RPCA). However, when restricting the analysis to only GC-MS features, classification accuracy dropped significantly with RPCA-ranked features (AUC-ROC = 0.65 ± 0.08), whereas Joint-RPCA substantially improved performance (AUC-ROC = 0.88 ± 0.04) (Fig. 5E).

Furthermore, we found that models trained on the full set of GC-MS features achieved classification performance comparable to models trained on small subsets of GC-MS features, but only when these features were resolved in the context of other omic layers (Fig. 5F). This result underscores Joint-RPCA’s ability to leverage interdependencies across omic types, optimizing feature selection while maintaining predictive power.

These findings demonstrated that Joint-RPCA not only integrates multi-omic data effectively but also enhances signal detection from weaker omic types, improving classification accuracy and biological interpretation. By resolving features in the broader multi-omic context, Joint-RPCA provides a more comprehensive understanding of microbial ecosystem function than single-omic analyses alone.

## Discussion

Here we present Joint-RPCA, a computational approach for multi-omics dimensionality reduction that resolves shared sample variation across multiple facets of microbial ecosystems (e.g., microbial abundances, metabolite levels, transcriptomics) and ranks the biological features associated with that variation. To the best of our knowledge, few existing unsupervised methods jointly address the sparsity, high-dimensionality, and compositionality typical of microbiome multi-omics data while also scaling to more than two omic types across matched samples. Notably, we found Joint-RPCA to be over 100 times faster than MMvec (Morton et al, 2019) in reconstructing known relationships between microbes and metabolites while maintaining strong benchmarking accuracy. Because Joint-RPCA operates on multiple omic layers simultaneously, rather than restricting analysis to pairwise combinations, it enables a more computationally efficient and integrated representation of cross-modal relationships.

Joint-RPCA is designed to reveal low-dimensional signals across data modalities by resolving features from each modality in the context of the others. For example, when applied jointly to microbial sequencing and metabolomics data, Joint-RPCA prioritizes microbial and metabolic features that together contribute to shared axes of variation, rather than selecting features independently within each modality. In data-driven simulations, Joint-RPCA showed stronger performance than current beta-diversity metrics and the benchmarked general-purpose multi-omics methods under sparse and compositional data conditions characteristic of microbiome datasets. Importantly, these methods were not all developed to address such data properties, and the observed performance differences should therefore be interpreted in that context rather than as a general statement about their overall utility.

In real data applications, Joint-RPCA demonstrated robustness and broad applicability across diverse biological contexts. When applied to the Integrative Human Microbiome Project (iHMP) dataset (Integrative HMP (iHMP) Research Network Consortium, 2014; Lloyd-Price et al, 2019), Joint-RPCA successfully distinguished inflammatory bowel disease (IBD) from non-IBD subjects across multiple omic layers, identifying cross-modal disease markers that were not apparent in single-omic analyses. This signal was replicated across two validation cohorts (Mills et al, 2022). Additionally, Joint-RPCA uncovered a universal decomposer network (Burcham et al, 2024) in microbial communities associated with human cadaver decomposition, integrating 16S and 18S rRNA sequencing, shotgun metagenomics, and metabolomics data to reveal shared ecological signatures across different environments. In a mammalian gut microbiome study (Gregor et al, 2022), Joint-RPCA enhanced classification performance of host characteristics by effectively leveraging weaker omic signals, such as gas chromatography–mass spectrometry (GC-MS) metabolomics, through joint factorization. Across these applications, Joint-RPCA provided an integrated view of cross-modal relationships and, in several settings, improved phenotype resolution relative to analyses performed on single omic types alone.

These findings underscore the value of multi-omics data and of methods tailored to the statistical characteristics of sparse, compositional biological datasets. By jointly factorizing multiple data modalities, Joint-RPCA uncovers shared low-dimensional structure across omic layers, capturing cross-modal relationships that may be overlooked in single-modality analyses. More broadly, these results support the value of integration strategies that are aligned with the statistical properties of the data being analyzed.

Several considerations are important when applying Joint-RPCA. First, Joint-RPCA relies on the assumption of a shared low-dimensional structure across data modalities. This assumption can be violated, for example, when the dominant structure in the data reflects a continuous gradient rather than a shared low-dimensional signal of interest (e.g., in continental-scale soil datasets, where the microbial ecosystem is strongly structured by soil pH (Lauber et al, 2009; Morton et al, 2017)). Our implementation of Joint-RPCA estimates the underlying rank and informs the user when the data may not meet this requirement (Keshavan et al, 2009). Second, as with other beta-diversity metrics, Joint-RPCA does not explicitly adjust for potential confounding variables that may affect downstream clustering, and therefore, additional validation is required. Third, while our framework can capture shared structure across data modalities, its performance can depend on the relative dimensionality and signal strength of each input dataset, a limitation that is common to many dimensionality reduction approaches. Differences in the number of features across omics may influence their relative contribution to the inferred joint components, requiring prior feature selection as suggested by prior studies (Helmer et al, 2024). Finally, when applying Joint-RPCA to longitudinal multi-omics datasets, researchers should note that temporal dynamics are not directly modeled, and therefore, the effect of time may be captured in addition to the phenotypic separation of interest. In such cases, we recommend complementary downstream analyses to separate the effects of time.

Our benchmarking strategy combined data-driven simulations, anchored in real microbiome multi-omics data with known phenotype labels, and in silico simulations with a designed low-dimensional structure. These settings allowed us to evaluate Joint-RPCA under realistic sparsity and compositionality conditions, as well as in a controlled setting with an imposed shared signal. Importantly, in the data-driven simulations, the true low-rank structure of the data, as well as the appropriate signal-noise decomposition, remains unknown, so performance can only be assessed indirectly through proxies such as phenotype separation in low-dimensional space. Conversely, the synthetic simulations are necessarily simpler than real biological systems and do not capture the full extent of biological and technical variability. Accordingly, our results should be interpreted as evidence that Joint-RPCA performs well in the specific scenarios studied, particularly when shared low-dimensional structure is present across modalities, rather than as evidence of universal superiority. In other datasets, especially those whose properties align more closely with the modeling assumptions of alternative methods, methods such as MOFA+ may perform better.

Finally, we acknowledge the recent contributions of web-based platforms such as Wekemo Bioincloud, which have facilitated the analysis of single- and multi-omics datasets through domain-specific tools and workflows (Gao et al, 2024). Additional comparisons against these platforms were not included, as they incorporate analytical frameworks that were individually tested against Joint-RPCA, including PCA- and PLS-based dimensionality reduction and beta-diversity analysis. For inferring cross-modal feature correlations, other popular methods such as ggClusterNet 2 have expanded co-occurrence analysis to accommodate multi-omics datasets (Wen et al, 2025). Importantly, their underlying computational frameworks primarily rely on correlation-based inference (e.g., Pearson, Spearman, or SparCC (Friedman and Alm, 2012)), which were also represented in our benchmarking analyses.

In summary, Joint-RPCA leverages the power of multi-omics study design and, specifically, the relationships across data modalities within an ecosystem to elucidate biological changes while reducing noise and increasing statistical power. We propose this tool both for the reanalysis of existing datasets and for future multi-omics studies across microbial and other biological systems in which sparsity, compositionality, and cross-modal structure are relevant.

## Methods

**Table:** Reagents and tools table

Reagent/resource	Reference or source	Identifier
**Software**		
**Python v. 3.9.19**	https://www.python.org/	
qiime2 v. 2024.5.1	https://qiime2.org/	
gemelli v. 0.0.12	https://github.com/biocore/gemelli	
MMvec (qiime2 v. 2019.10)	https://qiime2.org/	
scikit-bio v. 0.5.9	Aton et al, 2026	
scikit-learn v. 1.4.2	Pedregosa et al, 2011	
scipy v. 1.10.1	Virtanen et al, 2020	
seaborn v. 0.11.2	Waskom, 2021	
statannotations v. 0.6.0	Charlier et al, 2022	
pandas v. 1.5.3	The pandas development team, 2023	
matplotlib 3.8.4	Hunter, 2007	
mofax v. 0.3.6	https://github.com/bioFAM/mofax	
**R v. 4.3.3**	https://cran.r-project.org/	
phyloseq v. 1.52.0	McMurdie and Holmes, 2013	
SpiecEasi v. 1.99.0	Kurtz et al, 2015	
propr v. 5.0.4	Quinn et al, 2017	
mofa2 v. 1.18.0	Argelaguet et al, 2020	
mixOmics v. 6.32.0	Rohart et al, 2017	
iClusterPlus v. 1.44.0	Shen et al, 2009	
intNMF v. 1.3.0	Chalise and Fridley, 2017	

### Data preprocessing

We first identify the set of samples shared between all *K* input data modalities $\{{X}^{1},{X}^{2},\ldots ,{X}^{k}\}$. Samples with missing entries in at least one modality are removed from the joint factorization. Each matrix *X*^*k*^ is then transformed using the robust centered log-ratio (*rclr*) transformation to center the data around zero and approximate a normal distribution (Martino et al, 2019):

1 $${rclr}\left(x\right)=\left[\log \frac{{x}_{1}}{{g}_{r}\left(x\right)},...,\log \frac{{x}_{D}}{{g}_{r}\left(x\right)}\right]$$

where

2 $${g}_{r}\left(x\right)={\left({\prod}_{i\in {\Omega }_{x}}{x}_{i}\right)}^{1/|{\Omega }_{x}|}$$

*x*_*i*_ denotes the abundance of feature *i* (e.g., microbe, metabolite, or gene) in sample *x*, *D* is the number of features per modality, Ω_*x*_ is the set of features in sample *x* with nonzero (non-missing) abundances, and ${g}_{r}\left(x\right)$ is the geometric mean of features with abundance >0. Unlike the traditional *clr* transformation, *rclr* accommodates the sparsity often found in microbiome data without requiring imputation or the addition of pseudocounts. To perform cross-validation of the reconstruction error, the data can be split into train and test sets. The *rclr* transformation is then applied separately to each set, yielding: ${Y}_{{train}}^{k}={rclr}({X}_{{train}}^{k})$ and ${Y}_{{test}}^{k}={rclr}({X}_{{test}}^{k})$ (see Model cross-validation for details). For notation simplicity in the next section, we refer to ${Y}_{{train}}^{k}$ as *Y*^*k*^.

Importantly, Joint-RPCA does not require a unified preprocessing, normalization, or transformation scheme across modalities. Users may apply any preprocessing procedures suited to their data prior to integration. However, for compositional and highly sparse data modalities such as microbiome profiles, we recommend using the *rclr* transformation, which is implemented as the default transformation within Joint-RPCA’s framework.

### Joint-RPCA

Joint robust PCA (Joint-RPCA) extends the OptSpace matrix completion algorithm, which was originally designed to reconstruct highly sparse data under low-rank assumptions (Keshavan et al, 2009). As a dimensionality reduction algorithm, Joint-RPCA estimates shared low-dimensional factors that capture the principal sources of variation across modalities, while imputing missing entries. The objective function minimizes the reconstruction error defined by:

3 $$\begin{array}{c}{\min }_{{{{{\boldsymbol{U}}}}}_{{{{\boldsymbol{shared}}}}},{{{{\boldsymbol{V}}}}}_{{{{\boldsymbol{k}}}}}}\,\frac{{\sum }_{k=1}^{N}|\Lambda \left({{{{\boldsymbol{Y}}}}}^{{{{\boldsymbol{k}}}}}-{{{{\boldsymbol{U}}}}}_{{{{\boldsymbol{shared}}}}}{{{\boldsymbol{S}}}}{{{{\boldsymbol{V}}}}}^{{{{{\boldsymbol{k}}}}}^{{{{\boldsymbol{T}}}}}}\right){|}_{2}^{2}}{N}\end{array}$$

Where ${{{{\boldsymbol{U}}}}}_{{{{\bf{shared}}}}}$ is the sample loading matrix estimated jointly across all input matrices, ${{{{\boldsymbol{V}}}}}^{{{{\boldsymbol{k}}}}}$ is the feature loading matrix specific to modality *k*, ***S*** is the diagonal matrix of shared singular values, and Λ is an operator that masks missing entries, ensuring that the reconstruction error is computed only on observed entries. The optimization therefore generates shared latent factors ${{{{\boldsymbol{U}}}}}_{{{{\bf{shared}}}}}$ and ***S*** that minimize reconstruction error across all datasets simultaneously. Optimization is performed iteratively via gradient descent.

To ensure consistent rotation of the estimated matrices across iterations, the shared sample factors are orthogonalized at each step. Specifically, ${{{{\boldsymbol{U}}}}}_{{{{\bf{shared}}}}}$ and ***S*** are updated by recomputing a singular value decomposition (SVD):

4 $$\begin{array}{c}{{{{\boldsymbol{U}}}}}_{{{{\bf{shared}}}}}{{{\boldsymbol{S}}}}{{{{\boldsymbol{U}}}}}_{{{{\bf{shared}}}}}^{{{{\boldsymbol{T}}}}}={{{\mathrm{SVD}}}}\left(\frac{\mathop{\sum}_{k=1}^{N}{{{{\boldsymbol{U}}}}}^{{{{\boldsymbol{k}}}}}{{{{\boldsymbol{U}}}}}^{{{{{\boldsymbol{k}}}}}^{{{{\boldsymbol{T}}}}}}}{N}\right)\end{array}$$

where ${{{{\boldsymbol{U}}}}}^{{{{\boldsymbol{k}}}}}$ denotes the updated sample loadings matrix corresponding to modality *k* at that iteration. For each ${{{{\boldsymbol{V}}}}}^{{{{\boldsymbol{k}}}}}$ iteration update, we define an auxiliary matrix ${{{{\boldsymbol{W}}}}}^{{{{\boldsymbol{k}}}}}$ for each modality, given by:

5 $$\begin{array}{c}{{{{\boldsymbol{W}}}}}^{{{{\boldsymbol{k}}}}}={({{{\boldsymbol{S}}}}{{{{\boldsymbol{V}}}}}^{{{{{\boldsymbol{k}}}}}^{{{{\boldsymbol{T}}}}}})}^{T}\end{array}$$

For each modality, ${{{{\boldsymbol{W}}}}}^{{{{\boldsymbol{k}}}}}$ captures how strongly each feature contributes to the shared latent factors. Finally, for interpretability, the modality-specific feature loadings ${{{{\boldsymbol{V}}}}}^{{{{\boldsymbol{k}}}}}$ are concatenated into a single feature-by-component matrix, where features are grouped by modality but aligned along the same shared principal components. This unified representation allows users to rank features within and across modalities, and compare their relative contributions across each principal component.

Joint-RPCA applied to only one input matrix *X*^*k*^ is equivalent to the original RPCA (Martino et al, 2019) but with the additional benefit of cross-validation, which the original RPCA methodology lacked. While there is no strict upper limit for the number of data modalities *K* that Joint-RPCA can integrate, we recommend including modalities that are expected to contain meaningful and complementary biological signals.

#### Model cross-validation

To assess model generalizability, Joint-RPCA supports a cross-validation step whenever data are split into train and test sets. Users may provide their own train-test split; otherwise, Joint-RPCA automatically partitions the data internally. In either case, the objective function (Eq. 3) is optimized only on the train set. Test samples are projected into the latent space defined by the training matrices, and reconstruction error is calculated by:

6 $$\begin{array}{c}{cross}-{validation error}=\frac{\mathop{\sum }_{k=1}^{N}|\Lambda \left({{{{\boldsymbol{Y}}}}}_{{{{\boldsymbol{test}}}}}^{{{{\boldsymbol{k}}}}}-({{{{\boldsymbol{Y}}}}}_{{{{\boldsymbol{test}}}}}^{{{{\boldsymbol{k}}}}}\,{{{{\boldsymbol{V}}}}}_{{{{\boldsymbol{train}}}}}^{{{{\boldsymbol{k}}}}}){{{{\boldsymbol{W}}}}}_{{{{\boldsymbol{train}}}}}^{{{{\boldsymbol{T}}}}}\right){|}_{2}^{2}}{N}\end{array}$$

This allows users to evaluate whether the representation learned from the training data also minimizes error on unseen data. Furthermore, visualization of the cross-validation error over training iterations can be used to adjust model parameters (e.g., number of latent components) such that the error reaches a stable local minimum. Importantly, the number of iterations is defined by the user and is not tuned automatically. Thus, cross-validation serves as a post-hoc diagnostic tool, providing a quantitative metric that the learned representation generalizes across samples rather than overfitting to the training set. After the training is completed, samples from the test set are projected into the final latent space so that they are preserved for downstream analyses.

In addition to cross-validation, we highlight that Joint-RPCA includes an optional rank-estimation step that provides a data-driven suggestion for the number of latent components (*r*) based on the singular value spectrum and improvement in fit. This can further guide users when choosing this parameter in practice. Throughout our simulations and case studies, we primarily used two to three latent components because cross-validated performance plateaued beyond r=3. Increasing *r* did not materially improve held-out reconstruction error or downstream phenotype prediction, indicating that the dominant shared structure in these datasets is captured by the leading components.

#### Feature covariance

To quantify relationships between features across all input matrices, we compute a feature covariance matrix. First, for each modality *k*, we use the corresponding matrix ${{{{\boldsymbol{W}}}}}^{{{{\boldsymbol{k}}}}}$, which encodes the feature contributions to the shared latent factors. These matrices are concatenated (stacked) column-wise across modalities, and then multiplied by their transpose:

7 $${feature}\,{covariance}\,{matrix}=\left[\begin{array}{c}{{{{\boldsymbol{W}}}}}_{{{{\bf{1}}}}}\\ {{{{\boldsymbol{W}}}}}_{{{{\bf{2}}}}}\\ \vdots \\ {{{{\boldsymbol{W}}}}}_{{{{\boldsymbol{k}}}}}\end{array}\right]{\left[\begin{array}{c}{{{{\boldsymbol{W}}}}}_{{{{\bf{1}}}}}\\ {{{{\boldsymbol{W}}}}}_{{{{\bf{2}}}}}\\ \vdots \\ {{{{\boldsymbol{W}}}}}_{{{{\boldsymbol{k}}}}}\end{array}\right]}^{T}$$

This operation captures both within-modality and cross-modality covariance, providing a measure of how features jointly contribute to the shared latent structure identified by Joint-RPCA.

#### Terminology and modeling assumptions

In matrix factorization, low-rank means a structured component of the data is well-approximated by a small number of latent components; in many settings this corresponds to capturing a large share of structured variance, though the signal may still be small relative to total variation when embedded in high-rank noise. In microbiome settings, low-dimensional signal frequently refers to a biological effect that lies along a low-dimensional axis or factor (often rank-1 for two groups) even when it explains only a small fraction of total variance because it is embedded in high-rank biological and technical variation.

For example, a disease phenotype might manifest as a consistent shift in the abundance of a small subset of taxa (effectively one principal axis of differentiation), even if that axis is minor compared to other variability (e.g., interpersonal differences, temporal fluctuations). In such cases, the phenotype effect is a rank-1 mean shift (one latent factor) with sparse loadings, embedded in high-rank variation (Lloyd-Price et al, 2019). Mathematically, a matrix *X* with observed taxa abundances can be represented as $X=\mu +U{V}^{T}+E$, where *μ* captures mean taxa abundances, $U{V}^{T}$ captures the rank-1 phenotype effect, and *E* is full-rank noise. Therefore, we use low-dimensional signal to refer to the dimension of the latent phenotype factor, not the fraction of total variance it explains.

### Data-driven simulations

There is no gold standard or consensus on how to simulate microbiome sequencing data, especially in multi-omics studies, where there is a need to simulate multiple types of data modalities. Different frameworks make certain assumptions (zero-inflation, copulas, latent factors, etc.), and no consensus has emerged on one best way to simulate the complexity of microbial multi-omics data (Sankaran et al, 2024). To overcome this obstacle, we adopted a data-driven (semi-synthetic) simulation strategy, anchoring our simulations in real data distributions drawn from well-studied datasets, a commonly used approach in microbiome method evaluations that has been advocated to improve realism in microbiome benchmarking (Shenhav et al, 2019; Martino et al, 2021, 2019; Äijö et al, 2018; Morton et al, 2019; Quinn and Erb, 2021; Sankaran et al, 2024).

To benchmark Joint-RPCA using realistic microbiome data distribution and varying sparsity levels, we performed data-driven simulations using multi-omics data from the iHMP study (Lloyd-Price et al, 2019) and the biocrust soils study (Swenson et al, 2018) (see Datasets for data specifications). In both datasets, metagenomic taxonomic abundances were used as reference values for the simulations, while the remaining modalities were used in their observed form. Following the procedure of Quinn and Erb (Quinn and Erb, 2021), we generated metagenomic count tables by drawing from a multinomial distribution with predefined sequencing depths. Relative abundances from the original data served as sampling probabilities, ensuring that the empirical feature distribution and per-sample structure were preserved.

Specifically, data-driven simulated datasets were generated as follows:

1. **Sample selection**. Identify samples shared across all modalities to ensure alignment across data types. For the iHMP dataset, retain the last time point available per subject to capture the latest microbiome composition. This resulted in 68 shared samples from the iHMP data and 19 shared samples from the biocrust data.
2. **Simulation of metagenomic counts**. For each dataset (iHMP or biocrust), draw counts from a multinomial distribution ${x}_{i}\sim {{{\rm{Multinomial}}}}({d}_{i},{x}_{i})$, where ${p}_{i}$ are the feature-wise relative abundances for sample *i* and *d*_*i*_ is the specified sequencing depth. This preserves the original compositional structure of the data while inducing sparsity proportional to *d*_*i*_.
  - *iHMP data*. Depths of ${d}_{i}=67,000$, 16,000, 4000, 1000, and 250 were used to generate datasets with approximated densities of 11, 9, 7, 5, and 3%, respectively. The original metagenomics data was 12% dense, and other modalities were kept as observed.
  - *Biocrust data*. For each sample, the total observed count was divided by a scaling coefficient to set the effective sequencing depth *d*_*i*_. Coefficients of 270 and 2150 were used to produce densities of 1 and 10%, respectively. The original metagenomics data were 50% dense. To decrease the density of the metabolomics data from 99 to 90%, we used a scaling coefficient of $1* {10}^{5}$.
3. **Train-test splits**. For each sparsity level, data were partitioned into train-test splits. For consistency, the same splits were used across sparsity levels. These splits were used for methods capable of projecting unseen test data into the learned latent space (Joint-RPCA, MOFA+ (Argelaguet et al, 2020), and MMvec (Morton et al, 2019)).

- *iHMP data*. Ten independent random 75/25 train-test partitions using stratified sampling by diagnosis to preserve class balance. These splits were used to assess the stability of model performance and generalizability across different sample partitions.
- *Biocrust data*. Biocrust samples at intermediate successional stages (early-mid and late-mid) were assigned to the test set, while the remaining samples (early and late) were used for training. This time-resolved split assessed each model’s capacity to project unseen intermediate samples into the latent space.

4. **Data transformation**. Prior to downstream analyses, counts were transformed from the simplex (the constrained sample space of compositional data) to Euclidean space to enable Euclidean-based statistical analyses.

- *Joint-RPCA*. *rclr* transformation was applied to both iHMP and biocrust data.
- *MOFA*+, *iClusterPlus*, *intNMF*, and *multiblock sPLS* (iHMP data). *clr* transformation was used with a pseudocount of 1 to avoid zero division. This choice was motivated by the fact that iClusterPlus (Shen et al, 2009) and intNMF (Chalise and Fridley, 2017) cannot accommodate missing values, while MOFA+ (Argelaguet et al, 2020) and multiblock sPLS (Rohart et al, 2017) would otherwise ignore them, leading to potential information loss. To assess the impact of normalization choice, MOFA+ was additionally trained on the full iHMP data (no train-test splits) with *rclr* transformation. The resulting latent structure confirmed that normalization choice had minimal effect and was not the primary factor driving performance differences among methods.

c. Correlation-based methods (Biocrust data). Correlation-based metrics (i.e., Pearson, *ϕ*, Spearman, and *ρ*) were performed on *clr*-transformed data with the minimum nonzero value as the pseudocount, following the methods in Quinn and Erb (Quinn and Erb, 2021) through the propr package (Quinn et al, 2017) in addition to SPIEC-EASI (Kurtz et al, 2015), and SparCC (Friedman and Alm, 2012).

Importantly, random draws were not performed with fixed seeds, and therefore exact numerical results may vary slightly across independent runs. However, we anticipate qualitative outcomes and methods comparisons to remain stable across repetitions and provide all scripts necessary to reproduce the steps outlined above.

### Benchmarks for data-driven simulations

#### iHMP

The iHMP dataset was chosen due to its well-established IBD-associated signals across data layers. Lloyd-Price et al (Lloyd-Price et al, 2019) showed that this dataset harbors a low-dimensional phenotypic (IBD vs. non-IBD) signal that is statistically significant and biologically meaningful. To benchmark dimensionality reduction methods on their ability to recover this low-dimensional structure, each method was run with n=3 latent components for consistency. For Joint-RPCA and MOFA+ (Argelaguet et al, 2020), the data were partitioned into train and test sets, since both methods support projection of unseen samples into the learned latent space (see Data-driven simulations). While Joint-RPCA incorporates all modalities jointly during projection of held-out samples into ${{{{\boldsymbol{U}}}}}_{{\mbox{shared}}}$, MOFA+ performs this projection modality-by-modality, as outlined in github.com/bioFAM/mofax/blob/dev/docs/source/notebooks/project_data.ipynb. iClusterPlus (Shen et al, 2009), intNMF (Chalise and Fridley, 2017), and multiblock sPLS (Rohart et al, 2017) do not readily support out-of-sample projection; therefore, these methods were trained on the full dataset.

Phenotype separation in the inferred latent spaces was evaluated using complementary metrics computed on the sample loadings from each method (e.g., ${{{{\boldsymbol{U}}}}}_{{\mbox{shared}}}$ for Joint-RPCA). Wilcoxon rank-sum tests were performed independently for each PC or latent factor using scipy (v.1.10.1) (Virtanen et al, 2020). Mahalanobis distance between-group centroids was computed using a custom script. Briefly, distances were calculated as $d=\sqrt{{({{{{\boldsymbol{\mu }}}}}_{1}-{{{{\boldsymbol{\mu }}}}}_{2})}^{{{{\rm{\top }}}}}{{{{\boldsymbol{\Sigma }}}}}_{p}^{-1}({{{{\boldsymbol{\mu }}}}}_{1}-{{{{\boldsymbol{\mu }}}}}_{2})}$, where ${{{{\boldsymbol{\mu }}}}}_{1}$ and ${{{{\boldsymbol{\mu }}}}}_{2}$ are the cluster centroids and ${{{{\boldsymbol{\Sigma }}}}}_{p}=\frac{{{{{\boldsymbol{\Sigma }}}}}_{1}+{{{{\boldsymbol{\Sigma }}}}}_{2}}{2}+\lambda {{{\boldsymbol{I}}}}$ is the pooled covariance matrix, regularized by a small constant $\left(\lambda ={10}^{-6}\right)$ for numerical stability. PERMANOVA pseudo-F statistics were computed from pairwise sample distances using scikit-bio (v.0.5.9) (Aton et al, 2026). Random Forest (RF) classifiers were trained and evaluated using consistent train-test splits across all methods (see Data-driven simulations). For MOFA+, RF classifiers were trained and evaluated separately on each modality-specific projection, with performance metrics averaged across these projections. This framework ensured that the same splits were used across methods, while accommodating differences in each method’s support for the projection of unseen data. RF classification performance was assessed through the area under the receiver operating characteristic curve (AUC-ROC) and average precision-recall (APR) at each fold using scikit-learn (v.1.4.2) (Pedregosa et al, 2011).

#### Biocrust soils

To assess how accurately different computational methods recover experimentally validated microbe-metabolite interactions, we benchmarked several correlation- and model-based methods using data from the biocrust soils study (Swenson et al, 2018), which included both in situ samples and cultured isolates such as *M. vaginatus*. Because 70% of previously reported microbe-metabolite associations in this dataset were experimentally validated, these relationships were used as ground truth to evaluate each method’s accuracy. Specifically, we focused on associations between *M. vaginatus* and a previously curated list of metabolites shown to be directly linked through metabolic exchange (Morton et al, 2019). For each benchmarked method (Joint-RPCA, MMvec (Morton et al, 2019), SparCC (Friedman & Alm, 2012), SPIEC-EASI (Kurtz et al, 2015), Pearson, Spearman, *ϕ*, and *ρ* (Quinn and Erb, 2021)), we generated microbe-metabolite association matrices using the data-driven simulated datasets described above (see Data-driven simulations). Specifically, we compared results across three versions of the metagenomics data with different levels of sparsity: (i) the original counts (50% dense), (ii) an intermediately sparse version (10% dense), and (iii) a highly sparse version (1% density).

Performance was evaluated by ranking all possible microbe-metabolite pairs according to their inferred association strength and defining the top-*k* pairs as predicted interactions for increasing values of *k*. These predictions were compared to the ground-truth interaction set to compute counts of true positives (TP), false positives (FP), true negatives (TN), and false negatives (FN). From these counts, we derived standard metrics (precision and recall) to quantify each method’s ability to prioritize biologically validated interactions across a range of *k* thresholds and sparsity regimes.

### Pure simulations

In addition to the data-driven approach, we generated synthetic datasets to explore the strengths and limits of Joint-RPCA under controlled conditions where the ground truth was known. These simulations enabled systematic evaluation of the method’s stability, discriminative power, and ability to recover shared low-dimensional signal across modalities. We followed the approach introduced by Martino et al (Martino et al, 2019, 2021) and others (Äijö et al, 2018) to simulate compositional datasets with adaptable sparsity and effect sizes in two contexts: Low-dimensional phenotype signal simulations and breakpoint simulations.

#### Rationale for the low-dimensional phenotype signal simulations

In microbiome settings, low-dimensional signal frequently refers to a phenotype effect that lies along a low-dimensional axis (often rank-1 for two groups) even when it explains only a small fraction of total variance because it is embedded in high-rank biological and technical variation. For example, a phenotype may manifest as a consistent shift in the abundance of a small subset of taxa—effectively a single principal axis of differentiation—even if that axis is minor relative to other sources of variability (e.g., interpersonal differences, temporal fluctuations). In such cases, the phenotype effect can be represented as a rank-1 mean shift (one latent factor) with sparse loadings, embedded in high-rank variation. Here, we use low-dimensional signal to refer to the dimensionality of the latent phenotype factor, not the fraction of total variance it explains.

In the low-dimensional signal simulations described below, we therefore constructed a rank-1 latent factor (two groups) with sparse loadings (only a small subset of features in each modality carries the signal), embedded in high-dimensional noise. This design mirrors a common scenario in microbiome studies: a subtle phenotype effect (affecting relatively few microbial or molecular features) exists, while the data are dominated by other, unrelated variation. Mathematically, the data-generating model in our simulation comprises a rank-1 signal plus full-rank noise.

This simulation approach aligns with prior microbiome simulation frameworks that “spike in” low-dimensional case–control effects. For example, Sankaran et al (Sankaran et al, 2024) describe case–control simulators (e.g., MIDASim (He et al, 2024) and SparseDOSSA (Ma et al, 2021)) that generate group differences by allowing parameters for a subset of taxa to vary between cases and controls, with tunable effect sizes determined by the magnitude of parameter differences. Consistent with this paradigm, we progressively increased the number of differentiating features from 5 to 60 to modulate signal strength in a controlled manner. By holding the latent dimensionality fixed at 1 (two groups) and increasing the number of contributing features, we vary task difficulty, from very faint signals (5 features out of hundreds) to stronger signals (60 features), thereby assessing each method’s sensitivity to sparse, weak signals versus more distributed, stronger signals.

### Low-dimensional phenotype signal simulations procedure

To examine Joint-RPCA’s ability to recover such joint low-dimensional signals across data modalities with microbiome-like characteristics, we generated synthetic datasets as follows.

1. **Generate background distributions**. Three data matrices $\left({omi}{c}_{1}-{omi}{c}_{3}\right)$ were generated with n=50 samples and ${m}_{1}=100$, ${m}_{2}=150$, and ${m}_{3}=200$ features. Counts for each sample *i* and feature *j* are drawn from a normal distribution, ${s}_{{ij}}\sim N(0,1)$, to approximate microbiome-like data following *clr* or *rclr* transformations.
2. **Normalize to compositional data**. Values ${s}_{{ij}}$ were transformed to per-sample compositions by ${p}_{{ij}}=\frac{{s}_{{ij}}}{{\Sigma }_{j}{s}_{{ij}}}$ so that ${\sum }_{j}{p}_{{ij}}=1.$ This step constrains the data to the simplex, the sample space of compositional data.
3. **Add technical/biological noise and sparsity**. Compositions were transformed to Euclidean space using the additive log-ratio (ALR) transformation, ${ALR}({p}_{i}+c)$, with pseudocount *c* to avoid division by zero. This allows for the addition of normally distributed noise to mimic sequencing and biological variation. Next, values were mapped back to the simplex via the inverse ALR transformation and sparsity was introduced by setting a fraction of entries to zero, yielding datasets with a starting density of ~12% (Äijö et al, 2018). The ALR and inverse ALR transformations were implemented via skbio.stats.composition (Aton et al, 2026).
4. **Draw counts from a Poisson log-normal.** To approximate realistic sequencing data, compositional proportions ${p}_{{ij}}$ were converted into expected counts by ${\lambda }_{{ij}}={n}_{i}{p}_{{ij}}$ for each sample *i* with sequencing depth ${n}_{i}$ (i.e., total counts for sample *i*). Observed counts were then drawn from a Poisson log-normal (PLN) distribution, ${y}_{{ij}}={PLN}\left({\lambda }_{{ij}},\kappa \right)$, where *κ* controls the overdispersion of the underlying normal distribution. Finally, negative values were clipped to zero, and any all-zero rows or columns were removed (Martino et al, 2019, 2021).
5. **Introduce traceable signals.** To simulate low-rank structure, signal was induced in a subset of features, ${m}_{{induced}}$, for half of the samples $\left({n}_{{{\mathrm{induced}}}}=25\right)$ across all simulated modalities ${{{\mathrm{omic}}}}_{1}-{{{\mathrm{omic}}}}_{3}$. Values were drawn from a Poisson distribution with λ=1 and added to predefined entries, while the remaining unperturbed samples served as controls.
6. **Vary degree of induced signals.** The previous step was repeated while varying the number of features carrying induced signal (${m}_{{induced}}=5,\,10,\,20,\,30,\,40,\,50,$ and 60), resulting in progressively denser datasets. Across these conditions, data densities for ${{{\mathrm{omic}}}}_{1}-{{{\mathrm{omic}}}}_{3}$ ranged to ~12–14%, 13–15%, 14–18%, 15–20%, 17–23%, 18–26%, and 20–28%, respectively.
7. **Train-test splits.** For each simulation denoted by ${m}_{{{\mathrm{induced}}}}$, data were partitioned into ten independent 75/25 train-test splits stratified by groups with or without the induced signal to preserve class balance. For consistency, the same splits were used across ${m}_{{{\mathrm{induced}}}}$ simulations. These splits were used for methods capable of projecting unseen test data into the learned latent space (Joint-RPCA and MOFA+ (Argelaguet et al, 2020)).
8. **Data transformation**. Prior to downstream analyses, counts were transformed as described before:

- *Joint-RPCA*. *rclr* transformation was applied across simulated datasets.
- *MOFA*+, *iClusterPlus*, *intNMF*, and *multiblock sPLS*. *clr* transformation was used with a pseudocount of 0.1 to avoid zero division. This choice was motivated by the fact that iClusterPlus (Shen et al, 2009) and intNMF (Chalise and Fridley, 2017) cannot accommodate missing values, while MOFA+ (Argelaguet et al, 2020) and multiblock sPLS (Rohart et al, 2017) would otherwise ignore them, leading to potential information loss.

To ensure reproducibility, any steps requiring random draws were performed with ${{\mathrm{seed}}}=42$.

#### Breakpoint simulations

To evaluate how Joint-RPCA performs under varying effect sizes and feature-to-sample ratios, we simulated datasets where the separation between sample clusters gradually decreased. Smaller effect sizes correspond to stronger overlap between feature groups, which can challenge a method’s ability to recover distinct low-dimensional signals. We tested Joint-RPCA on pairs of simulated datasets (${omi}{c}_{1}$ and ${{{\mathrm{omic}}}}_{2}$) across three fixed data dimensionalities (n=20 samples and m=20,200, or 2,000 features). For each dimensionality, the feature overlap between sample clusters was increased from 10 to 50% in 10% increments. Each combination was repeated 15 times (folds) to generate technical replicates, and each replicate was run five times to assess stability.

Each synthetic dataset was generated as follows:

1. **Generate block-structured signals**. A block-diagonal matrix *S* was constructed, where each “block” corresponds to a sample cluster. In this matrix, each feature ${m}_{j}$ has a peak signal at ${\mu }_{j}$ and spreads along a sample gradient ${g}_{i}$ with standard deviation *σ*. This gradient represents phenotypic or environmental variation among samples. Entries in *S* are given by the Gaussian function: ${s}_{{ij}}=\frac{1}{\sqrt{2\pi {\sigma }^{2}}}exp \left(-\frac{{\left({g}_{i}-{\mu }_{j}\right)}^{2}}{2{\sigma }^{2}}\right)$. Adjacent blocks overlap based on a user-defined overlap parameter, which defines the degree of similarity between neighboring groups.
2. **Normalize to compositional data**. Values ${s}_{{ij}}$ were transformed to per-sample compositions by ${p}_{{ij}}=\frac{{s}_{{ij}}}{{\Sigma }_{j}{s}_{{ij}}}$ so that ${\sum }_{\,j}{p}_{{ij}}=1$. This step constrains the data to the simplex, the sample space of compositional data.
3. **Add noise and sparsity**. Compositions were transformed to Euclidean space using the additive log-ratio (ALR) transformation, ${{\mathrm{ALR}}}({p}_{i}+c)$, with pseudocount *c* to avoid division by zero. This allows for the addition of normally distributed noise to mimic sequencing and biological variation. Next, values were mapped back to the simplex via the inverse ALR transformation and sparsity was introduced by setting a fraction of entries to zero (Äijö et al, 2018). The ALR and inverse ALR transformations were implemented via skbio.stats.composition (Aton et al, 2026).
4. **Draw counts from a Poisson log-normal**. To approximate realistic sequencing data, compositional proportions ${p}_{{ij}}$ were converted into expected counts by ${\lambda }_{{ij}}={n}_{i}{p}_{{ij}}$ for each sample *i* with sequencing depth ${n}_{i}$ (i.e., total counts for sample *i*). Observed counts were then drawn from a Poisson log-normal (PLN) distribution, ${y}_{{ij}}={{\mathrm{PLN}}}\left({\lambda }_{{ij}},\kappa \right)$, where *κ* controls the overdispersion of the underlying normal distribution. Finally, negative values were clipped to zero, and any all-zero rows or columns were removed (Martino et al, 2019, 2021).
5. **Train-test splits**. For each simulated dataset, the first 25% of samples in each sample cluster were assigned to the test set, while the remaining samples were assigned to the training set. This ensured that the test set contained samples from all clusters, while the majority of samples remained in the training set.
6. **Data transformation**. Prior to downstream analyses with Joint-RPCA, counts were transformed with the *rclr* transformation.

For reproducibility, technical replicates were simulated with a random seed defined as $42+{{\mathrm{fold}}}+{{\mathrm{overlap}}}$.

#### Default parameters for the pure simulations

Throughout the pure simulations, the standard deviation was set to σ=2, the overdispersion parameter to κ=0.5, and the pseudocount to c=1. The latent sample gradient ${g}_{i}$ was defined as a linear spacing between 0 and 10, i.e., ${g}_{i}\in [0,10]$ for i=1,…,n, and represents continuous variation across samples. The feature-specific means ${\mu }_{j}$ are similarly spaced between 0 and 10 along the feature axis. Homoscedastic and heteroscedastic noise proportions are set to 0.5 each (i.e., 50% of counts are assigned random noise).

### Benchmarks for pure simulations

#### Low-dimensional phenotype signal simulations

To evaluate Joint-RPCA’s performance in identifying low-dimensional signals across sparse data modalities, we benchmarked it against MOFA+ (Argelaguet et al, 2020), iClusterPlus (Shen et al, 2009), intNMF (Chalise and Fridley, 2017), and Multiblock sPLS (Rohart et al, 2017) using simulated datasets described above. For each method, Random Forest classifiers were trained and tested on the estimated sample loadings across tenfold train-test splits. As described previously, projection of held-out test samples was performed only for Joint-RPCA and MOFA+, with MOFA+ projections computed separately for each modality (see Benchmarks for data-driven simulations). Next, we examined the feature ranks or weights along the principal component that best discriminated samples with induced signals from controls to verify whether the methods recovered the specific perturbed features. Among the benchmarked methods, only MOFA+ showed improved performance with an increasing number of features with signal, so feature rankings were compared between MOFA+ and Joint-RPCA. Each model’s feature selection performance was evaluated based on the number of true positives, as well as precision and recall, calculated among the top *k* ranked features or hits. Top *k* hits included both the highest- and lowest-ranked features to account for random directionality differences across runs (e.g., the top ten hits included the five highest-ranked plus the five lowest-ranked features).

#### Breakpoint simulations

RPCA (Martino et al, 2019) was applied independently to each simulated dataset, and Joint-RPCA was applied to pairwise combinations of simulated datasets with different effect sizes at a fixed dimensionality. For instance, for fixed dimensions (n=20 samples and m=200 features), Joint-RPCA was applied to ${omi}{c}_{1}$ and ${{{\mathrm{omic}}}}_{1}$, with ${{{\mathrm{omic}}}}_{2}$ anchored at a high effect size (small feature overlap between groups) and ${{{\mathrm{omic}}}}_{2}$ having increasingly smaller effect sizes (increasing number of overlapping features). Discriminative performance was quantified by PERMANOVA pseudo-F statistics computed from the sample loadings derived from either RPCA or Joint-RPCA. Consistency of feature loadings across independent runs of Joint-RPCA was assessed by computing Spearman correlations between the feature covariance matrices obtained across five independent folds.

### Runtime comparisons

For comparisons involving the FINRISK data (Borodulin et al, 2018), MMvec (Morton et al, 2019) and Joint-RPCA were run on a Intel(R) Core(TM) i7-8700 CPU @ 3.20 GHz (Architecture: x86-64, CPU max: 4.60 GHz, L1d cache: 32 K, L1i cache: 32 K, L2 cache: 256 K, L3 cache: 12288 K, RAM: 32GB, Disk: 2 TB SSD, Ubuntu 18.04.5 LTS, CPU(s): 12, Thread(s) per core: 2, Core(s) per socket: 6). For the comparisons involving the iHMP dataset (Lloyd-Price et al, 2019) and an independent IBD cohort (Mills et al, 2022), MMvec and Joint-RPCA were run on a Quad-Core Intel Core i7 computer (Architecture: x86-64, Total number of cores: 4, CPU max: 2.3 GHz, L2 Cache (per Core): 512KB, L3 Cache: 8MB, RAM: 16GB, Storage: 500GB, OS: Sonoma 14.7). Because MMvec takes as input two datasets at a time, it was run separately on all pairwise combinations of data modalities within each IBD dataset. To assess its scalability, we began with heavily subsampled feature sets and gradually increased the number of features until runtimes exceeded a practical threshold (>24 h). Beyond this point, runtimes were extrapolated by fitting an exponential function (via SciPy’s curvefit) (Virtanen et al, 2020) to the observed runtimes from completed runs, allowing us to extrapolate expected runtimes at larger sample sizes, up to the full dataset.

### Datasets

#### iHMP

Data modalities (i.e., metabolomics, metagenomics, metatranscriptomics, proteomics, and viromics) were obtained from https://ibdmdb.org/results. Before integration, samples shared across data modalities were selected, resulting in 135 samples (91 IBD and 44 non-IBD) containing 260 viral taxa, 8291 microbial pathways, 1471 bacterial taxa, 830 proteins, and 81,867 metabolites (Lloyd-Price et al, 2019). For data-driven simulations, subjects with multiple timepoints were subset to their last time point, resulting in 68 samples (49 IBD and 19 non-IBD).

#### Additional IBD cohorts

Three additional cohorts were included in order to demonstrate the replicability of results obtained with the iHMP cohort (Lloyd-Price et al, 2019). First, a cohort from UCSD that included 157 IBD patients and 16 healthy controls with shared multi-omic measurements: metaproteomics (108,080 features), metagenomics (3499 features), and metabolomics (1928 features) (Mills et al, 2022). Second, another UCSD cohort with 40 ulcerative colitis patients with 2572 metagenomic features (Mills et al, 2022). And third, 824 controls from the American Gut Project (AGP) with 10,010 metagenomic features from fecal samples (McDonald et al, 2018). Controls from the AGP were selected by excluding individuals with IBD or IBS, whether diagnosed by a medical professional, an alternative medicine practitioner, or self-diagnosed. Additionally, only individuals aged 25 years or older and without food allergies were included. Instructions for accessing the UCSD cohorts can be found at https://github.com/knightlab-analyses/uc-severity-multiomics. Specifically, processed data tables are available through MassIVE under accession MSV000086509. AGP sequence data and de-identified participant responses are available through the European Bioinformatics Institute under project PRJEB11419 and through Qiita (https://qiita.ucsd.edu/) under study ID 10317.

#### Biocrust soils

The data were retrieved from supplementary materials of the original publication (Swenson et al, 2018) and include 19 samples comprising 466 microbial taxa and 85 metabolites. True-positive metabolite associations and evaluation code were obtained from the Morton et al code repository (https://github.com/knightlab-analyses/multiomic-cooccurrences/tree/rebuttal) (Morton et al, 2019).

#### FINRISK

Patient recruitment, sample collection, and molecular processing are described in detail in the flagship publications (Borodulin et al, 2018; Salosensaari et al, 2021). The data supporting the runtime benchmarks were obtained from the THL Biobank through a written application in accordance with relevant Finnish legislation. Details on the application process are available at https://thl.fi/en/our-services/thl-biobank1/how-to-apply-for-thl-biobank-data-and-samples.

#### Mammalian gut

This dataset contains 101 fecal samples from 25 mammalian species, with three parallel data modalities: (1) 16S amplicon sequencing; (2) liquid chromatography tandem–mass spectrometry (LC–MS/MS); (3) gas chromatography–mass spectrometry (GC–MS) and gas chromatography-flame ionization detector (GC-FID) (Gregor et al, 2022). Following preprocessing as described in Gregor et al (Gregor et al, 2022), the data modalities comprised 8,153 bacterial taxa, 411 small, polar metabolites, 19,855 semi-polar metabolites, 8794 negative lipids, and 8036 positive lipids. The GC-MS data was filtered such that each feature had a balance score greater than 50. Raw sequencing data is available in the NCBI Sequence Read Archive under project PRJNA693262. The semi-polar metabolomics, GC-MS, and polar lipidomics data are available through MassIVE under accession numbers MSV000086131, MSV000083859, and MSV000091324, respectively.

#### Universal decomposer network

All modalities were obtained from https://github.com/Metcalf-Lab/2023-Universal-microbial-decomposer-network/tree/master/jrpca_network/network. This dataset comprises multi-omic measurements from 36 human cadavers, collected across four seasons from three different facilities (ARF, STAFS, and FIRS) (Burcham et al, 2024). For our analysis, we retained only hip-adjacent soil samples with data available from all omic types, resulting in 374 samples from 23 cadavers. These samples comprised 2333 metabolites, 5473 bacterial taxa from 18S and 14,237 from 16S, 257 bacterial genomes, 2457 gene abundances, and 377 gene modules.

### Quantitative methods for case studies

#### IBD cohorts

To identify features most strongly associated with the latent factors inferred by RPCA and Joint-RPCA, we ranked features within each data modality according to their loadings on the first principal components. For each dataset, the top and bottom features were defined as those with the largest positive and negative loadings, respectively, up to a modality-specific range. Specifically, we used 10–100 features for most data modalities, 2–10 for the virome, and 500–100,000 for the metatranscriptome to account for differences in feature dimensionality. To compute aggregate abundance of high- and low-loading features, we first added a small constant equal to the minimum nonzero abundance to each feature table to avoid division by zero. We then calculated, for each sample, a log-ratio of the total abundance of top-ranked features to that of bottom-ranked features. These log-ratios were used to validate the IBD structure captured along the first principal components of RPCA and Joint-RPCA, and to later confirm these IBD-related trends across independent cohorts (Lloyd-Price et al, 2019; Mills et al, 2022; McDonald et al, 2018).

#### Mammalian gut microbiomes

RPCA and Joint-RPCA were run on the available sample-matched tables with tenfold cross-validation of training and testing splits stratified by the lineage and digestive strategy of each mammal sampled (Gregor et al, 2022). RF multi-class classification was performed on the ten Joint-RPCA or individual RPCA folds with *OneVsRestClassifier* (Pedregosa et al, 2011). We benchmarked classification performance across three settings. First, RF classifiers were trained on all features from each modality separately to obtain a baseline estimate of the signal strength in each data modality, as measured by the area under the precision-recall curve (APR). Second, we trained RFs using only the top 5% features ranked by Joint-RPCA or RPCA. Top-ranked features from all modalities were combined and used as predictors, and we further evaluated models restricted to top-ranked metabolomics features only to test whether contextual information from other data modalities (captured by Joint-RPCA but not RPCA) improved their discriminative power. Third, we repeated this analysis using an increasing number of top metabolites (5–20) and observed that Joint-RPCA consistently identified metabolites with higher predictive accuracy compared to RPCA.

#### Universal decomposer network

We compared the sample loadings derived from individual RPCA and Joint-RPCA using the Kruskal–Wallis H-statistic from SciPy (v.1.10.1) (Virtanen et al, 2020), grouping samples by season, facility, or stage of decomposition. To identify the axis that best captures the decomposition stage, we employed the formula ${H}_{{{\mathrm{stage}}}}-({H}_{{{\mathrm{season}}}}+{H}_{{{\mathrm{facility}}}})$, where *H*_*x*_ represents the H-statistic for group *x*. The universal decomposer network was then generated following the approach by Burcham et al (Burcham et al, 2024), and Venn diagrams were used to evaluate the degree of overlap between RPCA- and Joint-RPCA-ranked features for each data modality. RF regression with *LeaveOneGroupOut* cross-validation (Pedregosa et al, 2011) was performed with ordination results from individual RPCA and Joint-RPCA. To limit the effects of facility and season in our model’s predictions, we trained and tested the RFs with samples from the same facility. Cross-validation mean absolute errors (MAE) were then calculated to assess RF performance.

## Supplementary information

Appendix

Peer Review File

Dataset EV1

Dataset EV2

Source data Fig. 2

Source data Fig. 3

Source data Fig. 4

Source data Fig. 5

Appendix Source Data

## Data Availability

The code generated in this study is available in the following repositories: -Analysis scripts: Code Ocean (https://doi.org/10.24433/CO.7782051.v1) -Analysis scripts: GitHub (https://github.com/Shenhav-Lab/Joint-RPCA-Manuscript/) The Joint-RPCA codebase, gemelli, is a fully unit-tested BSD-3 licensed open-source Python package and is installable through pip or conda. An R implementation is also available through the mia Bioconductor package (https://bioconductor.org/packages/mia). Additionally, Joint-RPCA is wrapped in a QIIME2 plugin (https://github.com/biocore/gemelli) and is available through this plugin in the Galaxy platform (Galaxy Community, 2024). A detailed tutorial illustrating the full workflow, recommended parameter settings, and integration with QIIME2 is provided in gemelli’s codebase (https://github.com/biocore/gemelli/ipynb/tutorials/Joint-RPCA-QIIME2-API.ipynb). All datasets analyzed in this study are publicly available, and information for data access is provided in the Methods section. The source data of this paper are collected in the following database record: biostudies:S-SCDT-10_1038-S44320-026-00236-3.
