# Supplementary material for: Joint-RPCA: domain-aware multi-omics integration for systems microbiology
Source: Mol Syst Biol. 2026 Aug 18;22(11):1789–808. doi: 10.1038/s44320-026-00236-3 (PMC13728224; doi:10.1038/s44320-026-00236-3)
Supplement: Supplementary file 1 [file 44320_2026_236_MOESM1_ESM.pdf]

Appendix for Joint-RPCA: Domain-Aware Multi-Omics Integration for Systems Microbiology

Table of Contents

Appendix Supplementary Figures. **2**

Appendix Figure S1. Dimensionality reduction of individual iHMP data types. *2*

Appendix Figure S2. Joint-RPCA yields more stable projection of unseen data to latent space and more consistent signal recovery across folds than benchmarked methods. *4*

Appendix Figure S3. Joint-RPCA more consistently recovers induced low-dimensional signals than the benchmarked methods. *5*

Appendix Figure S4. Simulation benchmarking of potential breakpoints. *6*

Appendix Figure S5. Biocrust benchmarking with intermediate density levels. *7*

Appendix Figure S6. Independent RPCA log-ratios (iHMP). *7*

Appendix Figure S7. Universal decomposer network associated with advanced stages of decomposition. *8*

Appendix Figure S8. Independent RPCA dimensionality reduction of human decomposition data. *9*

Appendix Figure S9. AUC-ROC curves for each omic type in the mammalian gut dataset. *9*

Appendix Supplementary Tables **10**

Appendix Table S1. Statistical comparison of RF metrics across folds for Joint-RPCA vs. benchmarks (iHMP data-driven simulations). *10*

Appendix Table S2. Statistical comparison of RF metrics across folds for Joint-RPCA vs. benchmarks (Low-dimensional phenotype signal simulations). *11*

Appendix Table S3. M. vaginatus relative abundances across biocrust samples. *12*

Appendix Table S4. M. vaginatus metabolite covariances across data densities. *13*

Appendix Table S5. Runtime comparisons of MMvec and Joint-RPCA (FINRISK). *14*

Appendix Supplementary Note: Comparison of Multi-Omics Dimensionality Reduction Methods Not Designed for Microbiome Data **15**

Appendix Supplementary Figures.

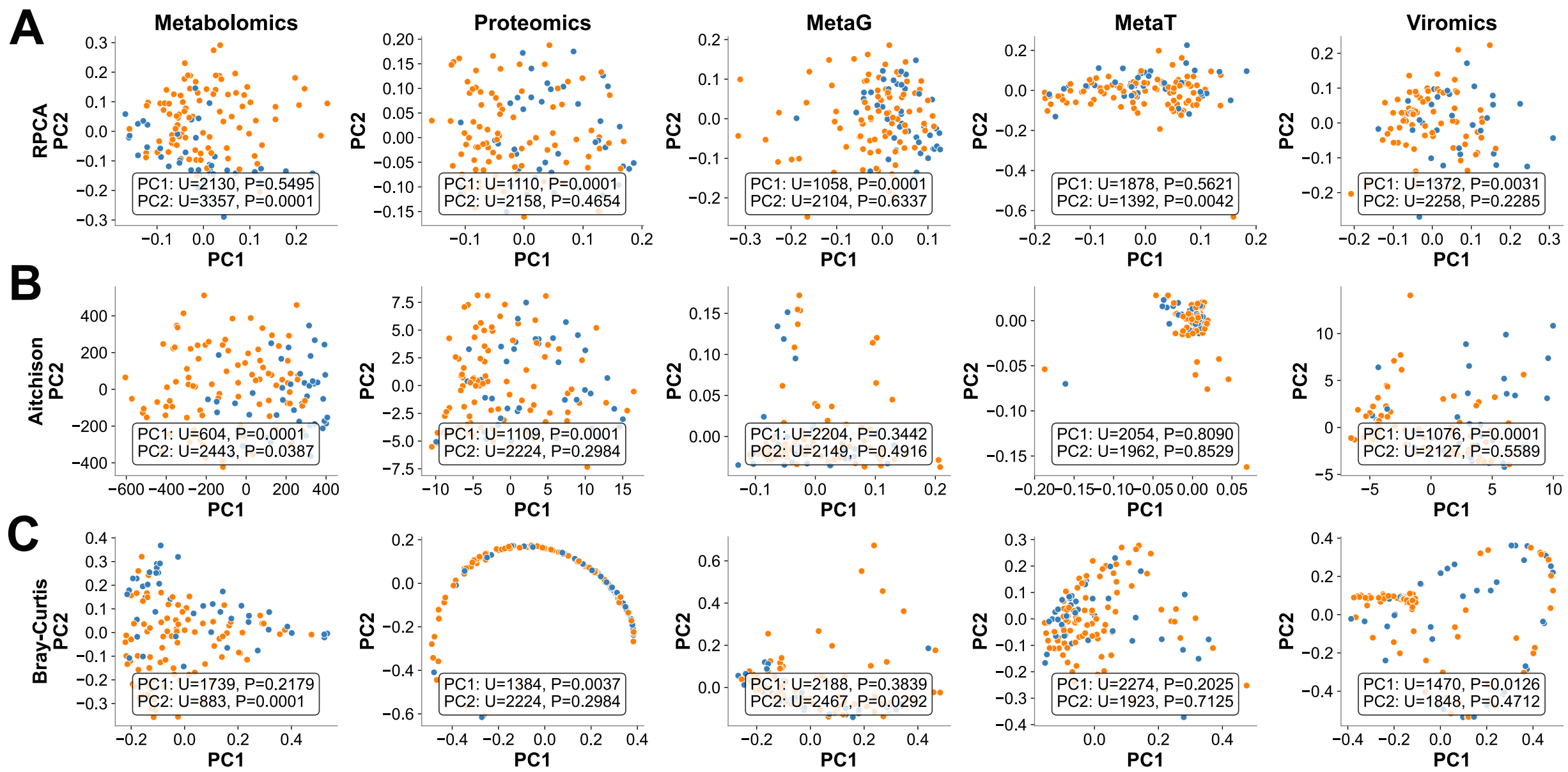

Appendix Figure S1. Dimensionality reduction of individual iHMP data types.

(A-C) Metabolomics, proteomics, metagenomics (MetaG), metatranscriptomics (MetaT), and viromics from the iHMP data, colored by IBD (n = 91, orange) vs. non-IBD (n = 44, blue) diagnosis were independently analyzed using RPCA **(A)**, and distance metrics Aitchison **(B)** and Bray-Curtis **(C)**. Statistical significance was assessed using the Wilcoxon rank-sum test; statistics and associated *p*-values are shown.

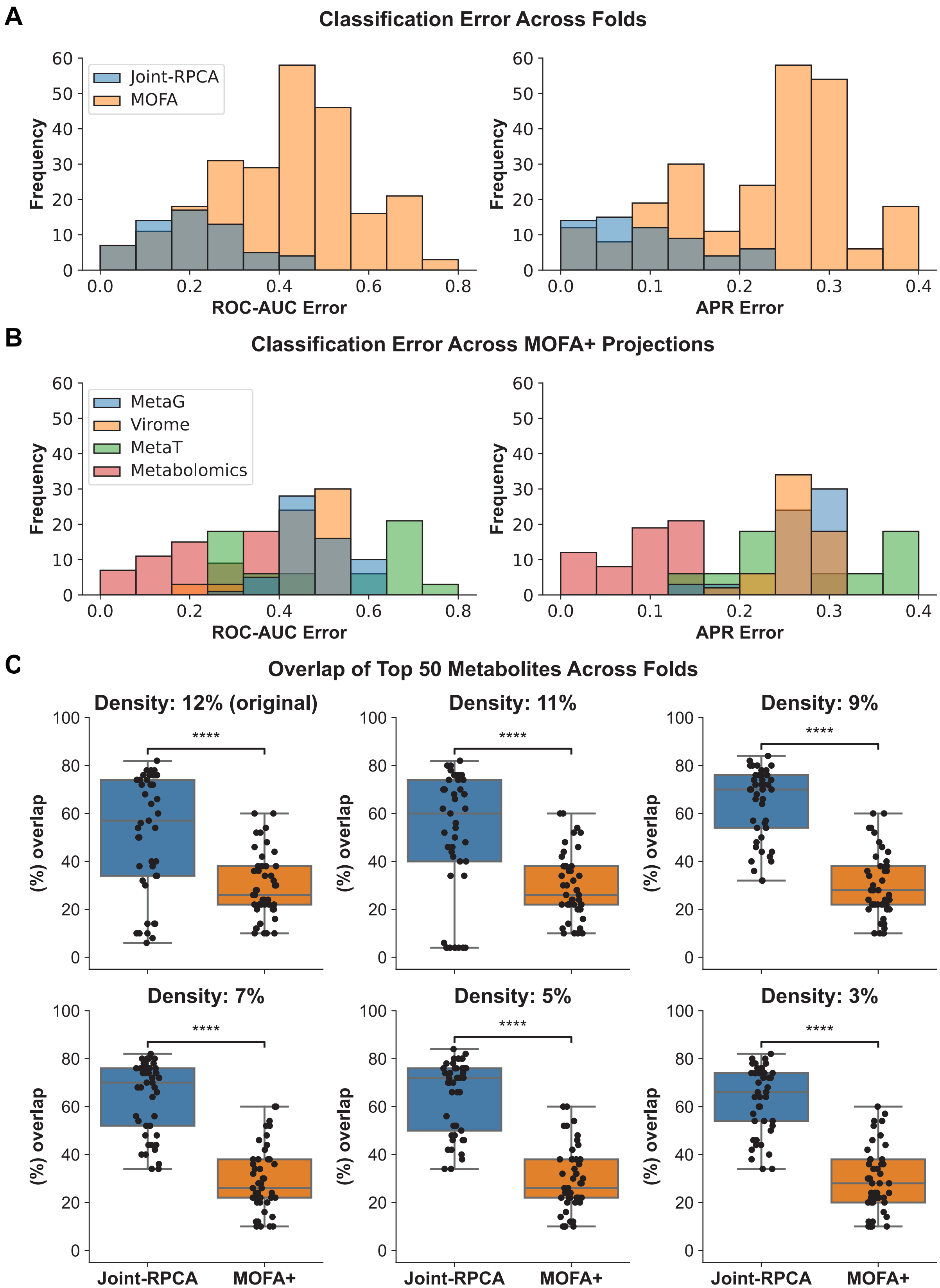

A
Classification Error Across Folds
Joint-RPCA
MOFA
Frequency
ROC-AUC Error
APR Error
B
Classification Error Across MOFA+ Projections
MetaG
Virome
MetaT
Metabolomics
Frequency
ROC-AUC Error
APR Error
C
Overlap of Top 50 Metabolites Across Folds
Density: 12% (original)
Density: 11%
Density: 9%
Density: 7%
Density: 5%
Density: 3%
(%) overlap
****
Joint-RPCA
MOFA+

41

Appendix Figure S2. Joint-RPCA yields more stable projection of unseen data to latent space and more consistent signal recovery across folds than benchmarked methods.

(A) Comparison of Random Forest APR and ROC-AUC error distributions evaluated on projected test data from Joint-RPCA (blue) and MOFA+ (orange) across 10 folds. **(B)** Error distributions across 10 folds of MOFA+ modality-specific projections show high variability in performance depending on modality, with metabolomics achieving the best performance. **(C)** Zoom in on metabolomics, the most predictive modality in MOFA+. Overlap of top- and bottom-ranked metabolomics features (n = 50) between all possible combinations of 10-fold train-test splits for Joint-RPCA (blue) and MOFA+ (orange). Joint-RPCA consistently selects similar features across the train-test folds, regardless of density level (60%-70% median overlap), whereas MOFA+ selects different features across folds (< 30% median overlap). This difference in overlap is statistically significant across all scenarios tested. Statistical significance was assessed using the Wilcoxon rank-sum test (****: $p < 0.0001$). For each box plot, the center line indicates the median, the box bounds indicate the interquartile range (25th-75th percentiles), and the whiskers extend to the most extreme data points within 1.5× the interquartile range.

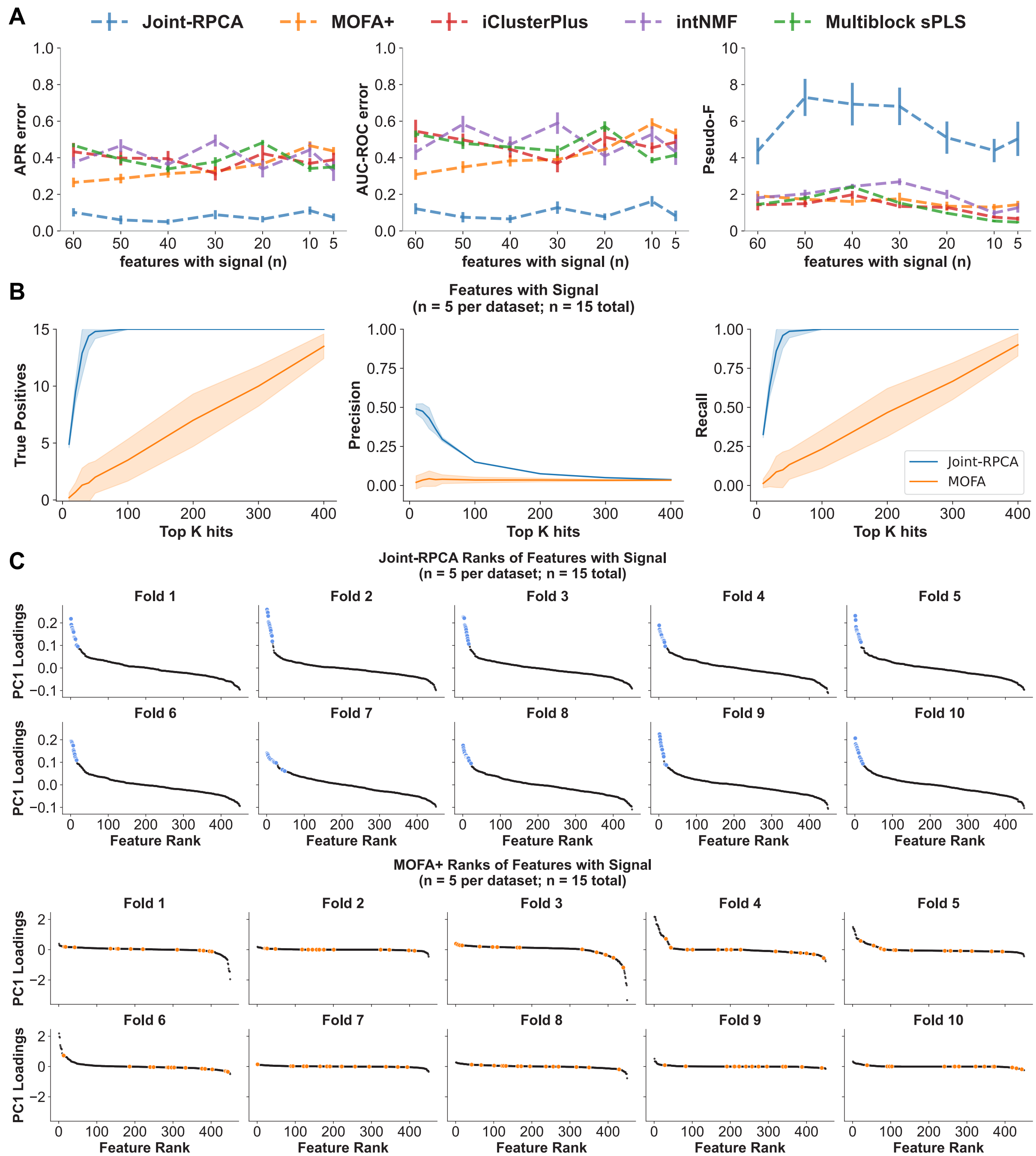

Appendix Figure S3. Joint-RPCA more consistently recovers induced low-dimensional signals than the benchmarked methods.

(A) Random Forest classification metrics (y-axis: APR error, left; AUC-ROC error, middle) and discriminative power (y axis: PERMANOVA pseudo-F, right) evaluated on unseen test samples, across 10-fold train-test splits. Three simulated datasets were used with an increasing number of features carrying induced signals that differentiate the groups (n = 5-60, x-axis in decreasing order). Error bars show the standard error of the mean across 10-fold cross-validation splits. **(B)** Recovery of induced features (n = 5 per dataset, n = 15 total) was assessed via true positives,

precision, and recall, among the top k ranked features (hits, x-axis) for Joint-RPCA (blue) and MOFA+ (orange). **(C)** Ranking (x-axis) of induced group-discriminating features along the most discriminative latent axis identified by Joint-RPCA (top) or MOFA+ (bottom). Joint-RPCA reliably ranks all 15 induced features (5 per modality) within the top 20 across folds, whereas MOFA+ ranks them less consistently and lower. The same features were induced for each method but are colored here to match their corresponding method.

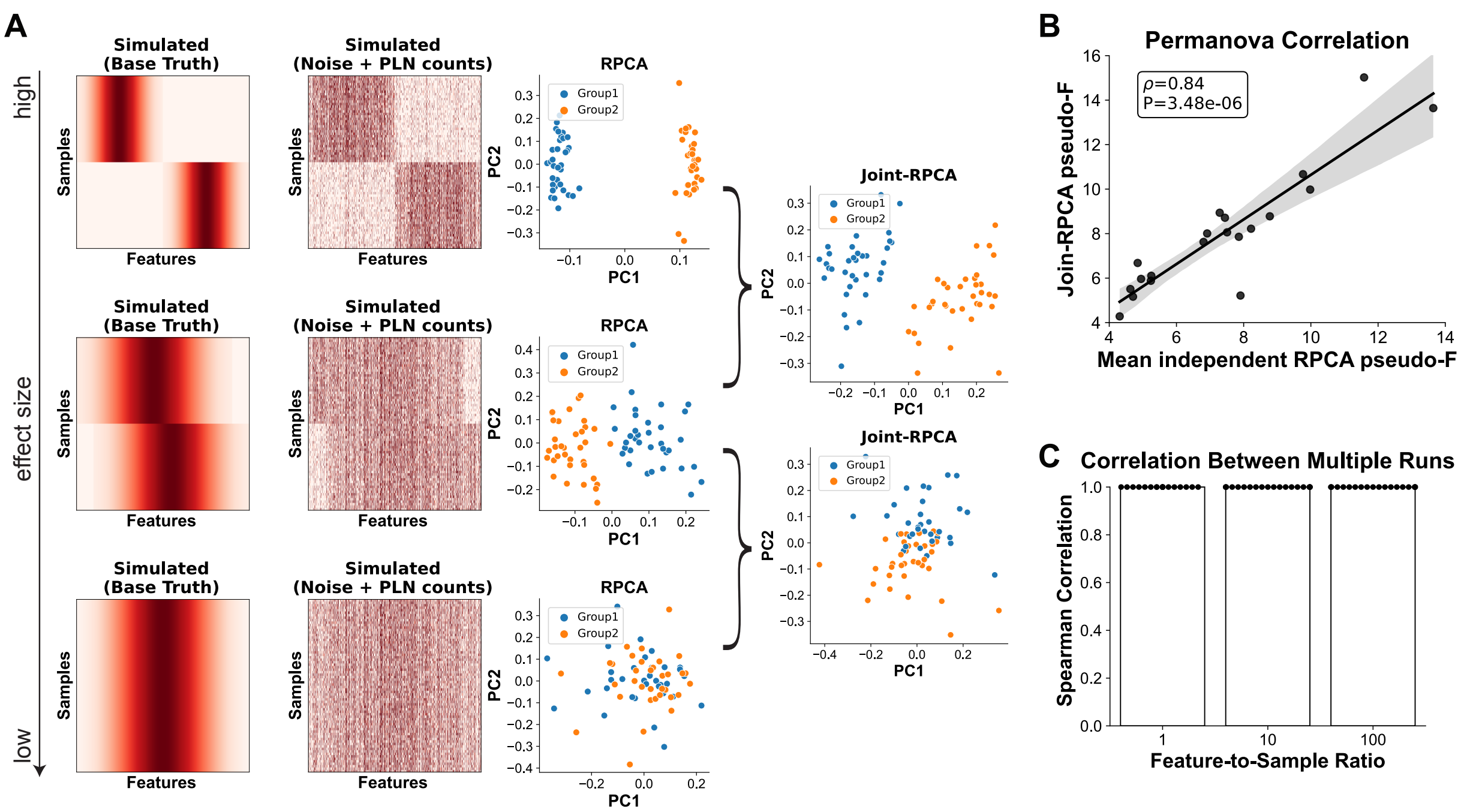

Appendix Figure S4. Simulation benchmarking of potential breakpoints.

(A) Simulations of three datasets with two sample groupings while varying their degree of feature overlap (i.e., effect size). RPCA is applied to independent datasets and Joint-RPCA to pairwise combinations to demonstrate underlying signal and discriminative power from each method. **(B)** Comparison of the PERMANOVA F-statistics (discriminative power) of the Joint-RPCA distance matrices (y-axis) by the mean independent RPCA across input simulation datasets (x-axis). **(C)** The Spearman correlation (y-axis) of Joint-RPCA sample and feature loadings across fivefold runs of each simulation pair for 1, 10, and 100 times as many features as samples (x-axis). The number of samples (n = 20) was consistent across simulations **(B-C)**. The number of features ranged from 20-2,000, depending on the feature-to-sample ratio used. The degree of feature overlap between phenotypic groups ranged from 10-50% in 10% increments, with 50% overlap corresponding to a noisy dataset with no sample groupings.

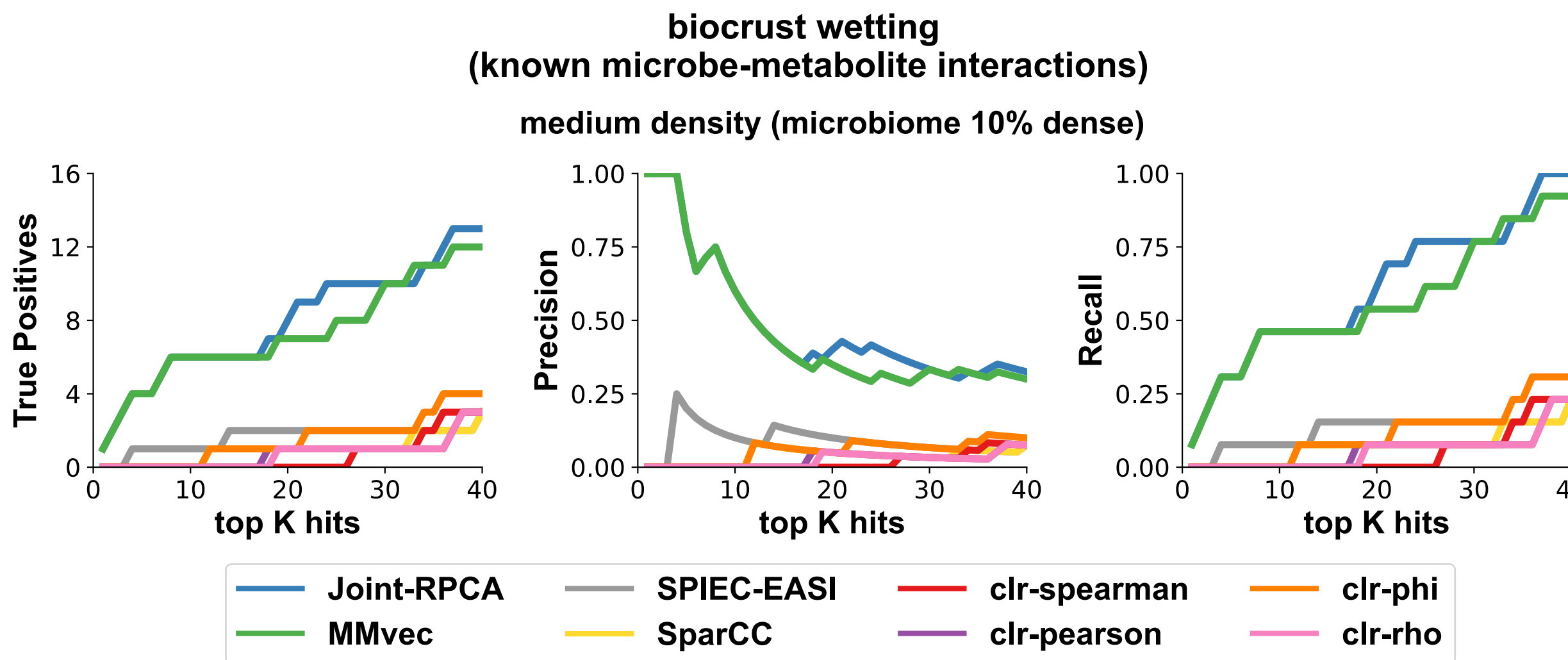

Appendix Figure S5. Biocrust benchmarking with intermediate density levels.

Benchmarking of association ranking of experimentally validated molecules of *M. vaginatus* in following biocrust wetting evaluated through true positive (left-column), precision (middle-column), and recall (right-column) (y-axes) in top K ranked hits (x-axes) on *in silico* subsampled sparse data (10% density) compared across methods Joint-RPCA (blue), MMvec (green), SPIEC-EASI (gray), SparCC (yellow), in addition to spearman (red), pearson (purple), phi (orange), and rho (pink) correlation metrics on clr-transformed data using imputation of the minimum non-zero value from each input table.

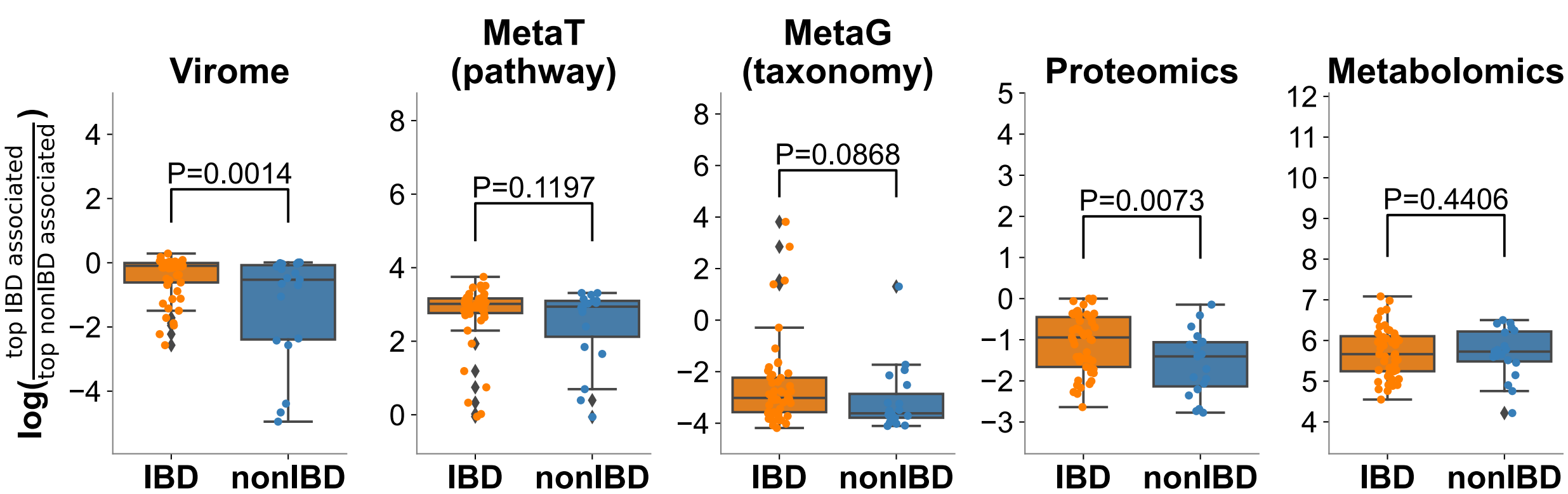

Appendix Figure S6. Independent RPCA log-ratios (iHMP).

Log-ratios of the top and bottom RPCA PC1-ranked features for each omic type in the iHMP cohort do not consistently distinguish between IBD (orange, n = 49) and non-IBD (blue, n = 19) patients. Statistical significance was assessed using an independent t-test; p-values are shown. For each box plot, the center line indicates the median, the box bounds indicate the interquartile range (25th-75th percentiles), and the whiskers extend to the most extreme data points within 1.5× the interquartile range.

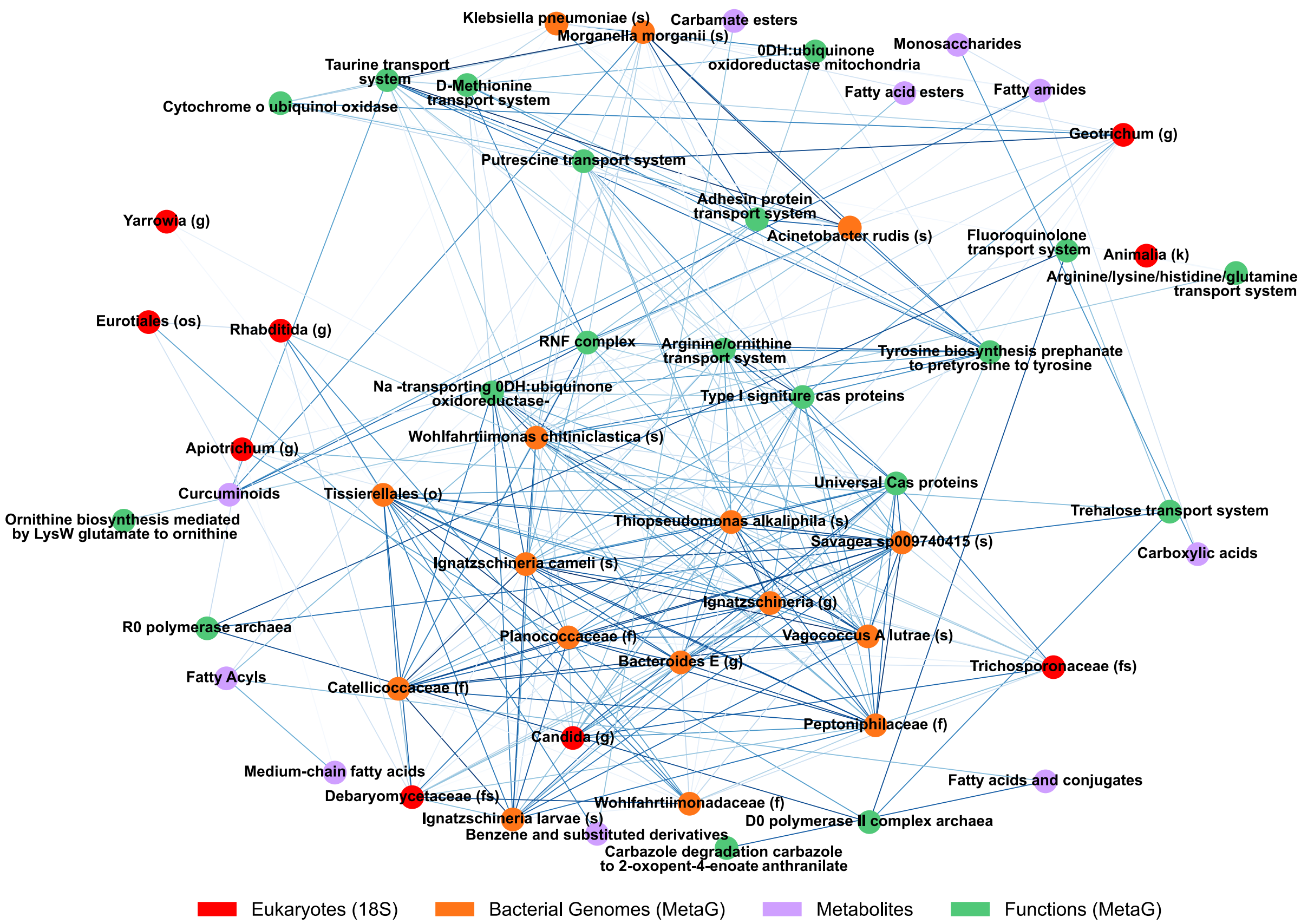

Appendix Figure S7. Universal decomposer network associated with advanced stages of decomposition.

The network showcases inter- and intra-domain relationships among features associated with advanced stages of decomposition. Briefly, top- and bottom-ranked features along Joint-RPCA PC2 loadings were selected for each omic type, and their log-ratios were used to determine decomposition directionality (i.e., whether top features were enriched in early vs. advanced stages of decomposition). The Joint-RPCA feature-feature correlation matrix of selected features served as the adjacency matrix to generate the network. Each node represents a feature, with edge weights indicating the strength of associations. Only the strongest correlations (>80th percentile of all absolute correlation values) are shown for clarity.

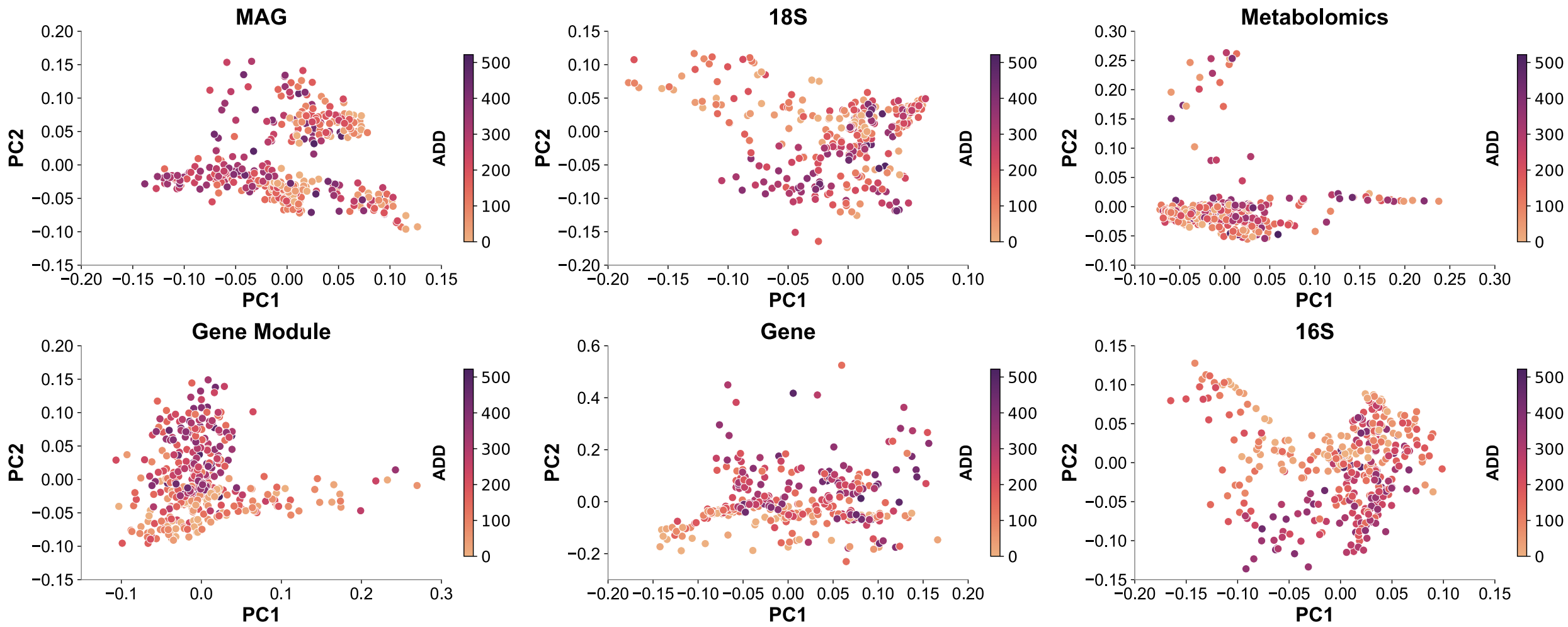

Appendix Figure S8. Independent RPCA dimensionality reduction of human decomposition data.

The progression of accumulated degree days (ADD) is not captured clearly with RPCA applied separately to each omic type.

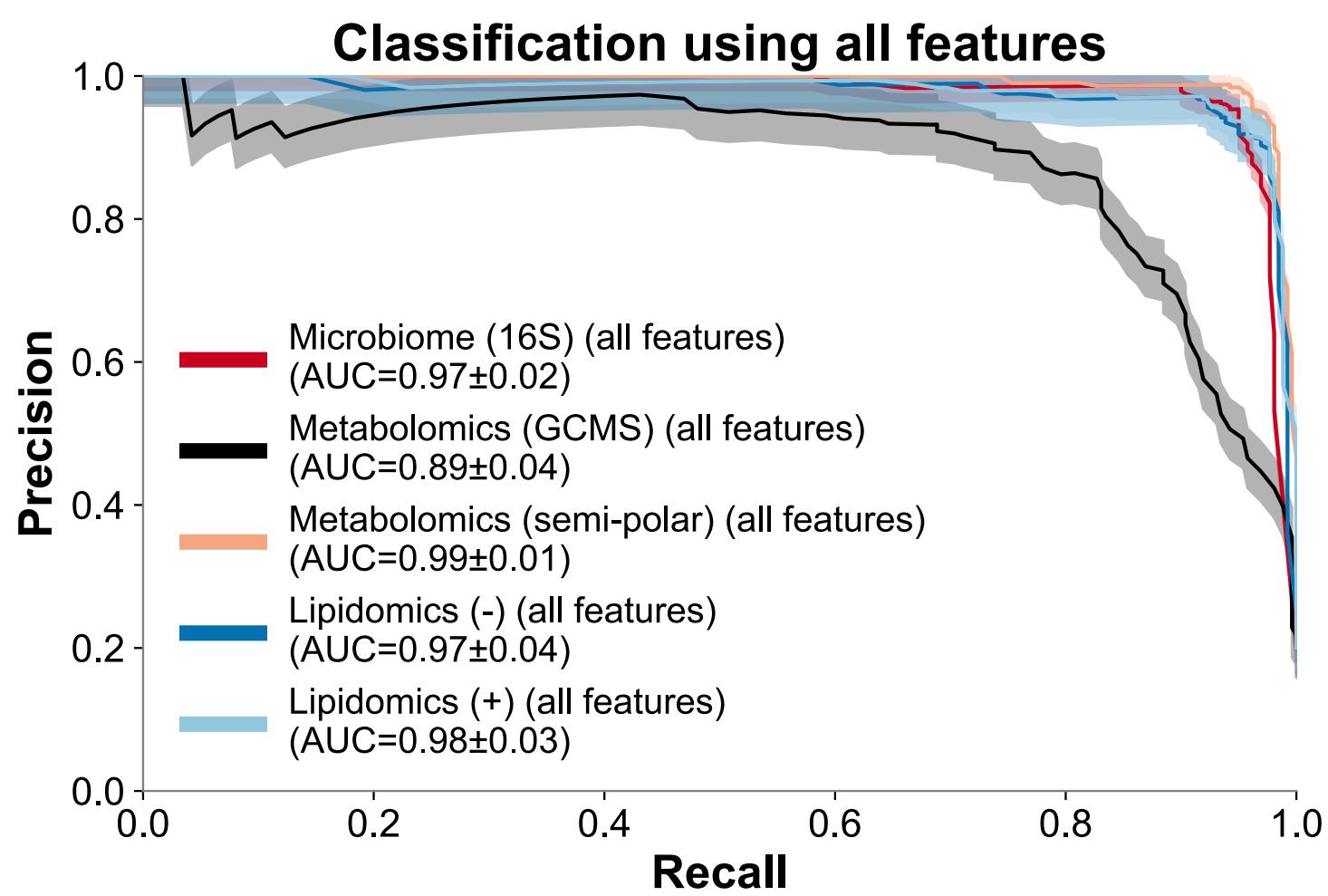

Appendix Figure S9. AUC-ROC curves for each omic type in the mammalian gut dataset.

AUC-ROC curves from Random Forest classifiers trained separately on all features from each omic type. Metabolomic (GC-MS) features have the lowest predictive performance for host lineage and digestive strategy compared to features from other omic types.

Appendix Supplementary Tables

Appendix Table S1. Statistical comparison of RF metrics across folds for Joint-RPCA vs. benchmarks (iHMP data-driven simulations).

MetaG Density	Comparison	Pseudo-F	Mahalanobis	APR Error	AUC-ROC Error
12%	Joint-RPCA vs. MOFA+	4.84E-06	4.05E-03	1.94E-04	8.74E-04
12%	Joint-RPCA vs. iClusterPlus	1.83E-04	1.83E-04	1.31E-03	2.19E-03
12%	Joint-RPCA vs. intNMF	1.83E-04	1.83E-04	1.31E-03	3.17E-03
12%	Joint-RPCA vs. Multiblock sPLS	1.83E-04	1.83E-04	1.01E-03	1.31E-03
11%	Joint-RPCA vs. MOFA+	3.41E-06	2.74E-03	8.37E-04	1.94E-03
11%	Joint-RPCA vs. iClusterPlus	1.83E-04	1.83E-04	1.01E-03	2.19E-03
11%	Joint-RPCA vs. intNMF	1.83E-04	1.83E-04	7.28E-03	3.59E-03
11%	Joint-RPCA vs. Multiblock sPLS	1.83E-04	1.83E-04	3.76E-02	2.83E-02
9%	Joint-RPCA vs. MOFA+	3.03E-06	7.91E-03	1.53E-03	2.13E-03
9%	Joint-RPCA vs. iClusterPlus	1.83E-04	1.83E-04	1.71E-03	2.79E-03
9%	Joint-RPCA vs. intNMF	1.83E-04	1.83E-04	4.59E-03	2.81E-03
9%	Joint-RPCA vs. Multiblock sPLS	1.83E-04	1.83E-04	1.13E-02	4.06E-03
5%	Joint-RPCA vs. MOFA+	3.03E-06	2.74E-03	1.09E-03	6.41E-04
5%	Joint-RPCA vs. iClusterPlus	1.83E-04	1.83E-04	2.83E-03	2.19E-03
5%	Joint-RPCA vs. intNMF	1.83E-04	1.83E-04	1.71E-03	7.65E-04
5%	Joint-RPCA vs. Multiblock sPLS	1.83E-04	1.83E-04	7.69E-04	5.80E-04
3%	Joint-RPCA vs. MOFA+	2.12E-06	4.38E-05	1.13E-04	1.66E-04
3%	Joint-RPCA vs. iClusterPlus	1.83E-04	1.83E-04	1.83E-04	3.26E-04
3%	Joint-RPCA vs. intNMF	1.83E-04	1.83E-04	4.40E-04	5.80E-04
3%	Joint-RPCA vs. Multiblock sPLS	1.83E-04	1.83E-04	4.40E-04	4.35E-04

P-values from two-sided Wilcoxon rank-sum test calculated on the 10-fold cross-validation sets.

138 **Appendix Table S2. Statistical comparison of RF metrics across folds for Joint-RPCA vs.**
139 **benchmarks (Low-dimensional phenotype signal simulations).**

N Induced	Comparison	APR Error	AUC-ROC Error	Pseudo-F
5 features	Joint-RPCA vs. MOFA+	6.37E-06	5.46E-06	2.14E-04
5 features	Joint-RPCA vs. iClusterPlus	2.45E-04	2.08E-04	3.30E-04
5 features	Joint-RPCA vs. intNMF	5.77E-04	5.75E-04	2.20E-03
5 features	Joint-RPCA vs. Multiblock sPLS	3.51E-06	6.72E-06	5.50E-06
10 features	Joint-RPCA vs. MOFA+	4.74E-06	3.76E-06	4.00E-05
10 features	Joint-RPCA vs. iClusterPlus	1.83E-04	1.78E-04	1.83E-04
10 features	Joint-RPCA vs. intNMF	2.46E-04	1.82E-04	1.83E-04
10 features	Joint-RPCA vs. Multiblock sPLS	9.87E-06	2.29E-05	3.01E-06
20 features	Joint-RPCA vs. MOFA+	1.52E-05	1.40E-05	2.31E-05
20 features	Joint-RPCA vs. iClusterPlus	2.46E-04	1.81E-04	1.83E-04
20 features	Joint-RPCA vs. intNMF	4.40E-04	7.48E-04	5.83E-04
20 features	Joint-RPCA vs. Multiblock sPLS	3.02E-06	2.97E-06	3.01E-06
30 features	Joint-RPCA vs. MOFA+	1.14E-04	2.00E-04	5.23E-05
30 features	Joint-RPCA vs. iClusterPlus	1.00E-03	2.19E-03	1.83E-04
30 features	Joint-RPCA vs. intNMF	1.82E-04	3.26E-04	1.71E-03
30 features	Joint-RPCA vs. Multiblock sPLS	1.75E-05	2.45E-05	5.50E-06
40 features	Joint-RPCA vs. MOFA+	7.36E-06	9.68E-06	1.75E-05
40 features	Joint-RPCA vs. iClusterPlus	1.79E-04	3.18E-04	7.69E-04
40 features	Joint-RPCA vs. intNMF	1.79E-04	1.78E-04	2.20E-03
40 features	Joint-RPCA vs. Multiblock sPLS	3.50E-06	3.71E-06	1.01E-04
50 features	Joint-RPCA vs. MOFA+	8.22E-05	1.61E-04	1.32E-05
50 features	Joint-RPCA vs. iClusterPlus	2.40E-04	2.29E-04	1.83E-04
50 features	Joint-RPCA vs. intNMF	1.78E-04	1.71E-04	2.46E-04
50 features	Joint-RPCA vs. Multiblock sPLS	6.35E-06	5.80E-06	4.08E-06
60 features	Joint-RPCA vs. MOFA+	1.88E-03	1.59E-03	1.52E-03
60 features	Joint-RPCA vs. iClusterPlus	4.40E-04	2.42E-04	1.71E-03
60 features	Joint-RPCA vs. intNMF	1.83E-04	4.97E-04	2.83E-03
60 features	Joint-RPCA vs. Multiblock sPLS	3.02E-06	3.37E-06	8.52E-06

P-values from two-sided Wilcoxon rank-sum test calculated on the 10-fold cross-validation sets.

Appendix Table S3. *M. vaginatus* relative abundances across biocrust samples.

Time	Biocrust Stage	Abundance (%)
3min	early	20.17
3min	earlymid	22.48
3min	latemid	9.68
3min	late	25.19
9hr	early	9.50
9hr	earlymid	7.19
9hr	latemid	9.16
18hr	early	14.16
18hr	earlymid	4.89
18hr	latemid	5.46
18hr	late	5.07
42hr	early	7.97
42hr	earlymid	6.51
42hr	latemid	0.49
42hr	late	3.82
49hr	early	1.21
49hr	earlymid	2.88
49hr	latemid	0.31
49hr	late	0.27

Appendix Table S4. *M. vaginatus* metabolite covariances across data densities.

original high density (microbiome 50% dense)

Rank	Metabolite	M. Vaginatus Covariance
1	adenine	5.64872
2	(N6-acetyl-lysine)	5.11957
3	4-guanidinobutanoate	4.79786
4	adenosine	4.14256
7	guanine	3.54506
8	cytosine	3.51027
17	uracil	2.16563
20	N-acetylornithine	1.73106
21	7-methyladenine	1.67476
23	(3-methyladenine)	1.46612
35	xanthine	0.95059
36	succinate	0.81901
39	hypoxanthine	0.79603

medium density (microbiome 10% dense)

Rank	Metabolite	M. Vaginatus Covariance
1	adenine	0.406624
2	(N6-acetyl-lysine)	0.365182
3	4-guanidinobutanoate	0.335393
4	adenosine	0.294940
7	cytosine	0.243281
8	guanine	0.239451
18	uracil	0.125318
20	N-acetylornithine	0.094299
21	7-methyladenine	0.082964
24	(3-methyladenine)	0.068231
34	hypoxanthine	0.023186
36	succinate	0.019722
37	xanthine	0.019649

low density (microbiome <1% dense)

Rank	Metabolite	M. Vaginatus Covariance
1	adenine	0.032147
2	(N6-acetyl-lysine)	0.028796
3	4-guanidinobutanoate	0.026261

4	adenosine	0.023954
7	cytosine	0.019825
8	guanine	0.018746
18	uracil	0.009857
20	N-acetylornithine	0.007452
24	(3-methyladenine)	0.005033
27	7-methyladenine	0.004710
33	succinate	0.001227
35	xanthine	0.001080
37	hypoxanthine	0.000672

Appendix Table S5. Runtime comparisons of MMvec and Joint-RPCA (FINRISK).

		time (seconds)		time (hours)	
**number samples**	**method**	**Simulation**	**FINRISK**	**Simulation**	**FINRISK**
50	Joint-RPCA	0.778	3.55	9E-06	4E-05
	MMvec	191.361	214.816	0.053	0.06
100	Joint-RPCA	1.334	7.163	0.0004	0.002
	MMvec	335.085	339.565	0.093	0.094
500	Joint-RPCA	7.938	47.483	0.002	0.013
	MMvec	1067.208	1546.913	0.296	0.43
1000	Joint-RPCA	15.836	79.653	0.004	0.022
	MMvec	1147.617	3799.261	0.319	1.055
6000 (all-samples)	Joint-RPCA	-	938.96	-	0.261
	MMvec	-	38600	-	10.722

Appendix Supplementary Note: Comparison of Multi-Omics Dimensionality Reduction Methods Not Designed for Microbiome Data

Multi-omics integration is crucial for understanding microbial ecosystems, yet existing methods face significant limitations when applied to microbiome data due to challenges such as sparsity and compositionality. Below, we outline the key features, computational approaches, and weaknesses of commonly used multi-omics dimensionality reduction techniques, emphasizing their constraints in the context of microbial community studies.

1. MOFA+ (Multi-Omics Factor Analysis)

Main Concept: MOFA is a probabilistic framework designed to extract latent factors that explain the shared and unique variation across multiple omic layers. It models multi-omics data by decomposing the input datasets into a lower-dimensional space using a factor analysis approach.

Computational Approach:

- MOFA employs a Bayesian hierarchical model to infer latent factors.
- It optimizes model parameters using variational inference techniques, making it scalable to large datasets.
- Regularization constraints help prevent overfitting and enhance interpretability.

Limitations for Microbiome Data:

- Assumes Gaussian distributions for continuous data and Poisson distributions for count data, which are not appropriate for microbiome (compositional) data.
- Struggles with high sparsity typical in microbial datasets.

2. iClusterPlus

Main Concept: iClusterPlus extends the iCluster framework by integrating multiple omics datasets using a latent variable approach. It primarily focuses on clustering by modeling the shared variance across different omic layers.

Computational Approach:

- Uses a Gaussian latent variable model to capture the correlation structure between omic layers.
- Employs K-means clustering on inferred latent variables for sample classification.
- Implements L1 and L2 regularization to control model complexity and enhance feature selection.

Limitations for Microbiome Data:

- Assumes normally distributed data, which is unsuitable for zero-inflated, compositional microbiome datasets.
- Performance may degrade when handling sparse omic layers.

3. IntNMF (Integrated Non-Negative Matrix Factorization)

Main Concept: IntNMF applies non-negative matrix factorization (NMF) to multi-omics data, identifying shared and data-specific components that contribute to variation across omic layers. It is primarily used for clustering and feature selection.

Computational Approach:

- Decomposes each omic dataset into two matrices: a shared feature matrix and a weight matrix.
- Uses multiplicative update rules to optimize the factorization while enforcing non-negativity.
- Can incorporate sparsity constraints to enhance interpretability by focusing on the most relevant features.

Limitations for Microbiome Data:

- Requires non-negative data, making it incompatible with log-ratio transformations commonly used in microbiome studies.
- Does not inherently account for the compositional nature of microbial datasets.
- Sensitive to noise and missing values, which are prevalent in microbiome studies.

4. Multiblock sPLS from MixOmics

Main Concept: MixOmics is a multivariate statistical framework designed to explore associations between different omic datasets. It provides both supervised and unsupervised approaches for dimensionality reduction and feature selection. Among available methods, we used **multiblock sPLS** to perform multi-omics integration.

Computational Approach:

- Multiblock sparse Partial Least Squares (sPLS) expands sPLS to multiple datasets with shared samples.
- Employs a design matrix to define relationships between omic datasets.
- Allows for sparse regularization, selecting the most informative features while reducing noise.

Limitations for Microbiome Data:

- Does not explicitly address the compositional structure of microbiome data, leading to potential biases in microbial feature selection.

5. Joint-RPCA: Addressing Microbiome-Specific Challenges

Main Concept: Joint-RPCA is specifically designed for multi-omics dimensionality reduction in microbial communities. It overcomes the limitations of traditional methods by explicitly handling data sparsity and respecting the compositional nature of microbiome data.

Computational Approach:

- Utilizes Robust Principal Component Analysis (RPCA) to decompose multi-omics data into low-rank components.
- Avoids Gaussian assumptions, making it well-suited for zero-inflated and non-Gaussian datasets.
- Incorporates constraints that respect the compositionality of microbiome data, ensuring biologically meaningful interpretations.
- Resolves features in the context of other modalities, enabling the discovery of biologically relevant patterns that drive host-associated phenotypes.

By integrating these considerations, Joint-RPCA offers a more biologically informed framework for multi-omics analysis in microbial community studies, overcoming the critical limitations of existing methods.
